# Optimizing pollencounter for high throughput phenotyping of pollen quality in tomatoes

**DOI:** 10.1016/j.mex.2020.100977

**Published:** 2020-06-27

**Authors:** Mathieu Anatole Tele Ayenan, Agyemang Danquah, Charles Ampomah-Dwamena, Peter Hanson, Isaac K. Asante, Eric Yirenkyi Danquah

**Affiliations:** a West Africa Centre for Crop Improvement, College of Basic and Applied Science, University of Ghana, Legon, Ghana; b The New Zealand Institute for Plant & Food Research Limited (PFR), Auckland, New Zealand; c World Vegetable Center, West and Central Africa – Coastal and Humid Regions, IITA-Benin Campus, Abomey-Calavi, Republic of Benin; d University of Ghana, Department of Plant Biology and Environmental Science, Legon, Ghana

**Keywords:** Heat-tolerance, ImageJ, Solanum lycopersicum, Pollen viability

## Abstract

The macro “PollenCounter” in ImageJ was initially developed to assess pollen viability in grapevine. We set out to see if PollenCounter could be used to assess pollen number and viability in tomatoes.

• We tested different optimization scenarios by adjusting the pollen size (100–900, 200–900 pixel^2^) and circularity of pollen grains (0.4–1, 0.5–1, and 0.6–1) on 31 microscopic images of stained tomato pollen. Both total pollen number and proportion of viable pollen were positively and significantly correlated with the outputs from manual counting. The scenario with 100–900 pixel^2^ pollen size and 0.4–1 circularity had the highest association for pollen number (*r* = 0.99) and pollen viability (*r* = 0.86). PollenCounter is 32-fold faster than manual counting.
• We added a command to the macro to automatically save the outputs containing the number of total and viable pollen, avoiding transcription errors inherent to manual counting.
• We successfully applied the optimized PollenCounter to discriminate tomato genotypes based on pollen number and pollen viability under heat stress. Our results show that PollenCounter, as an open-access macro, can be customized and improved to meet users’ needs. The use of PollenCounter can save time and money in pollen quality assessment. We outline the steps to optimize the macro for other samples or crop species. The optimized macro could allow efficient screening of a large germplasm collection for pollen thermo-tolerance and selection of best thermo-tolerant individuals in breeding programs.

## Graphical abstract

**Figure:**
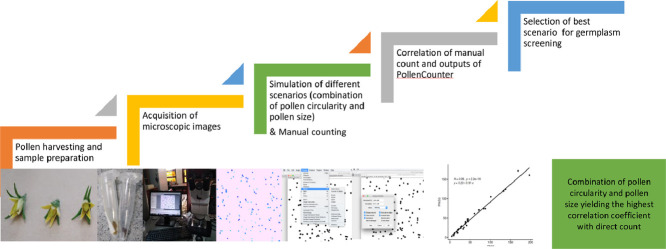

**Table:** Specifications Table

Subject Area	Agricultural and Biological Sciences
More specific subject area	Phenotyping
Method name	PollenCounter, an open-source ImageJ-based macro
Name and reference of original method	Tello J, Montemayor MI, Forneck A, Ibáñez J. A new image-based tool for the high throughput phenotyping of pollen viability: Evaluation of inter- and intra-cultivar diversity in grapevine. Plant Methods. 2018;14:1–17.
Resource availability	Necessary data to reproduce the method are included in the manuscript, and Supplementary materials and additional information.

## Introduction

Phenotyping has become the limiting factor for efficient trait discovery and deployment as the cost of genotyping is decreasing. The reliability of germplasm characterization and selection in programs is intrinsically linked to the quality of the phenotypic data. Phenotypic data acquisition and processing have been plagued by a lack of proper techniques [5,10].

Tomato production is adversely affected by heat stress and this situation is going to worsen [1]. It is urgent to leverage available technologies and advances in ‘omics’ and data science to fast-track the development of heat-tolerant tomato varieties. Pollen viability is a key component of plant tolerance to heat stress [1,4]. Assessing pollen thermo-tolerance is then critical for the identification of sources of tolerance to be used in breeding programs. The importance of assessing pollen viability has been subject to intensive research. However, quick and reliable techniques have limited the characterization of large germplasm collections and the identification of sources of pollen thermo-tolerance [4]. Current methods for pollen viability are either based on manual counting or are fully automated. The manual counting requires staining of pollen grains, visualization using light microscope and counting of viable and non-viable pollen on a haemocytometer based on different color patterns [8]. In addition to pollen viability, this method also allows estimation of the average pollen number per flower. However, manual counting is laborious and limits the numbers of accessions and samples that can be evaluated. Plus, it is prone to various errors including colors’ appreciation, counting, and data recording errors. Automated devices for pollen counting and estimation of pollen viability are available but they are not open-source. The cost of these devices can be too costly for breeding or research programs with a limited budget.

It is important to find reliable and accurate techniques that minimize the phenotyping cost, and are easily accessible for pollen viability assessment. Combining differences in pollen size and staining would increase the power and accuracy of estimating pollen viability. In this regard, Tello et al. [13] developed a semiautomatic technique including pollen staining, microscopic image acquisition, and processing with the macro “PollenCounter” in ImageJ [11]. PollenCounter requires the use of a staining dye called “modified Alexander dye” [7], where viable and non-viable pollen grains develop dark blue and light blue stains, respectively. The image processing is mainly based on particle counts in red and green channels indicating the total pollen number and the number of viable pollen on the microscopic image, respectively. PollenCounter was initially developed to assess pollen viability in grapevine and showed excellent capacity to accurately estimate pollen number and pollen viability [13]. Pollen size and shape, which is an important parameter of the macro, vary greatly among plant species [2,12]. Tello et al. [13] recommended that PollenCounter could be optimized for other crop species by modifying pollen size and circularity. Here, we optimized PollenCounter for high-throughput assessment of pollen viability in tomatoes.

## Method details

### Pollen harvesting and handling

Three to five newly opened flowers from the top of the plants were selected per genotype (see Additional information for more details on the genotypes). The sepals and petals and ovary of selected flowers were removed and the anther was sliced and put in a 1.5 ml Eppendorf tube. We used a modified Alexander staining dye [7], composed of Malachite green, Orange G, acid Fuschin, glycerol, acetic acid, distilled water, and 95% alcohol. 40 µl of the solution was added to the sliced anther and vortex for 20 s for pollen release. 10 µl of the solution was pipetted and loaded on a preheated slide. Pollen grains were viewed using a Leica DM500 compound microscope (objective 40X) with ICC50 camera and Leica LAS Software Version 2.1.0 (Leica Microsystems, Wetzlar, Germany). The viable pollen stained dark blue and dead pollen stained light blue [7]. Three to four images having a size of 2048 × 1536 pixels were taken per genotype. We prepared 31 images for the optimization of PollenCounter (Supplementary Materials).

### Image processing: optimization of the macro

**Fig. 1:**
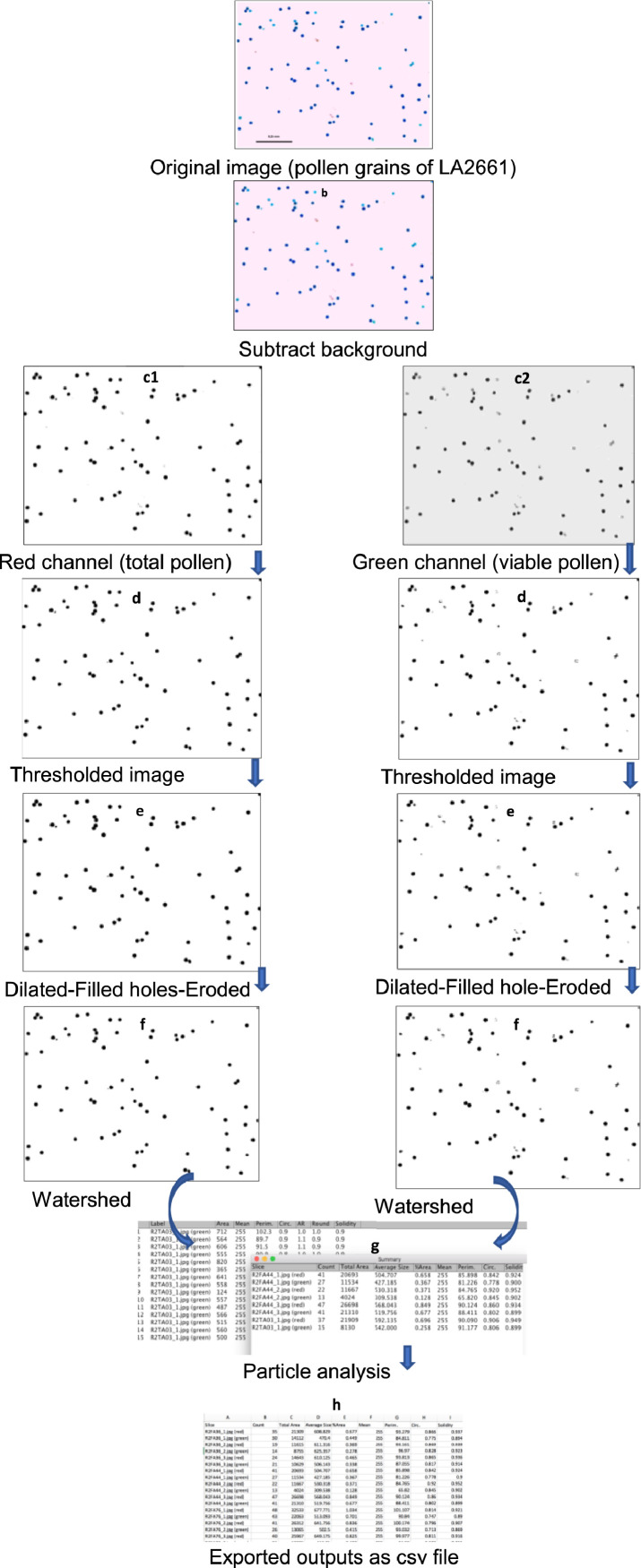
Adapted from Tello et al. [13]. Different image processing commands of PollenCounter from original image to export of outputs. (a) Input image with dark blue and light blue colored pollen grains, (b) output of subtraction of background from input image, (c) splitting image into red and green channels which are automatically saved; (d) converting images into binary image (black particles on a white background), (e) add pixels to edge of black particles, fill holes and remove pixels from edges of black particles, (g) separation of clustered particles of images, (h) Simultaneous count of particles in red and green channels, (g) Saving outputs in a .csv file in working directory (For interpretation of the references to color in this figure legend, the reader is referred to the web version of this article.).

PollenCounter consists of subtracting the image background, and splitting the images in red, green and blue channels and processing the red and the green channels. The red and green channels represent the total number of pollen and viable pollen grains respectively. The processing of these two channels involves thresholding to get 8-bit images, dilating, filling holes, eroding, and watershed. The watershed algorithm is used for segmentation and enables the separation of over-clustered pollen grains. The final step is the particle counting whereby the number of particles in the red and green channels is counted and the output is saved as a csv file (Fig. 1).

- *Manual counting*

The manual counting was done on the acquired images. We did a direct count of dark blue and light blue pollen grains on each image. Total pollen number and number of viable pollen were recorded. The percentage of viable pollen were computed as follows:

$$\text{PV}=\frac{Number\;of\;viable\;pollen}{Total\;pollen\;number}x100$$

- *Use of original macro*

We first used the original macro (S0) with the parameters, 60–800 for the red channel which records the total pollen number and 100–800 which records the number of viable pollen and circularity of 0.4–1 for both green and red channels [13]. This macro yielded more than 100% for pollen viability percentage for three images. This suggested that for some images, PollenCounter with the original parameters overestimated the number of viable pollen or underestimated total pollen number in tomatoes.

- *Optimization scenarios*

We selected a range of circularity (0.4–1) and pollen size (100–900 pixel^2^) to make sure all the pollen grains were measured. We combined two levels of pollen size, namely 100–900 and 200–900 pixel^2^ and three levels of circularity, namely 0.4–1, 0–5–1, 0.6–1 to form six scenarios (Table 1, Supplementary Materials). For each scenario, we used the same circularity and pollen size for total pollen number and viable pollen. The original PollenCounter scenario, S0, is defined by a pollen size of 60–800 pixel^2^ for total pollen number and 100–800 for viable pollen and circularity of 0.4–1 for both pollen types.

**Table 1:** Scenarios.

	100–900	200–900
0.4–1	S1 (100–900; 0.4–1)	S2 (200–900; 0.4–1)
0.5–1	S3 (100–900; 0.5–1)	S4 (200–900; 0.5–1)
0.6–1	S5 (100–900; 0.−61)	S6 (200–900; 0.6–1)

We assessed the correlation between total pollen grains and pollen viability on each image and manual count. The packages ggplot2 (Wickham et al. [14]) and ggpbur [3] in R (R [9]) were used for data visualization.

**Fig. 2:**
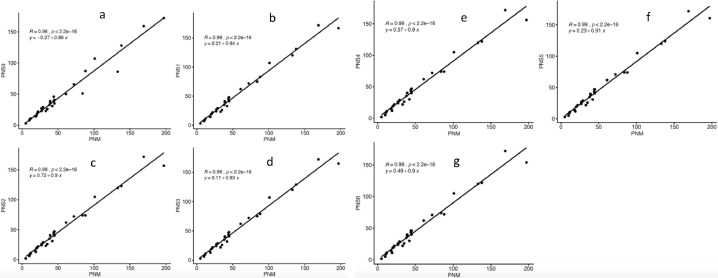
Prediction of automatic pollen number from manual pollen count.

**Fig. 3:**
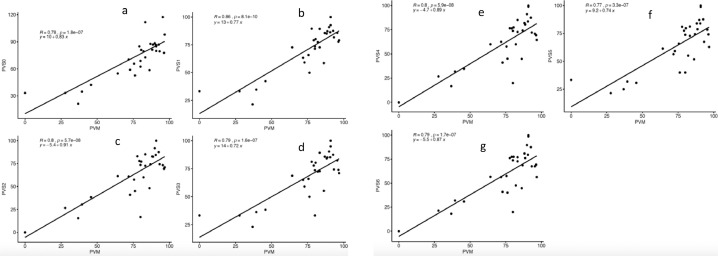
Prediction of automatic pollen viability from manual pollen count.

All the scenarios yielded positive and significant (*p*<0.001) association between outputs of manual counting for both total pollen number and proportion of viable pollen. The scenario S1 with 100–900 pixel^2^ pollen size and 0.4–1 circularity had the highest correlation for both pollen number (*r* = 0.99, *p*<0.001) and proportion of pollen viability (*r* = 0.86, *p*<0.001) (Fig. 2, Fig. 3) with manual count. Overall, the association between S1 pollen number and manual count was higher (0.98≤*r* ≤ 0.99) than that between S1 pollen viability (0.77≤*r* ≤ 0.86) and manual count. This suggests that the specified pollen size enabled the macro to capture most of the pollen grains. The association with the manual counting for the proportion of pollen viability was also excellent. Tello et al. [13] made similar observations regarding the high correlation between manual count and automatic count from PollenCounter in grapevine. Taken together, PollenCounter accurately estimated pollen number and pollen viability when optimized appropriately.

Time and money are limited resources in breeding programs. PollenCounter takes 3.4 ± 0.68 s to process one image (from image loading into ImageJ to the counting of total and viable pollen) compared to 108 ± 71 s for manual counting (counting of total and viable pollen grains). We added a command [saveAs(“Results”, “/Directory/filename.csv”);] to the original macro to automatically save the outputs containing the number of total and viable pollen in the working directory, avoiding transcription errors inherent to manual counting. PollenCounter makes it possible for images to be processed at the convenience of the users, eases data sharing, and can save time and money in pollen quality assessment.

The optimized macro offers the opportunity to efficiently screen a large germplasm collection for pollen thermo-tolerance and selection of the best thermo-tolerant individuals in breeding programs. Increased efficiency in phenotyping can ultimately be translated into increased genetic gain [10].

The use of PollenCounter for phenotyping pollen of a given sample need optimization. However, the time needed for the optimization is insignificant compared with labor-intensive manual counting. We outlined the different steps for quick and accurate optimization of PollenCounter.

### Good practices to increase the accuracy of pollencounter

**Fig. 4:**
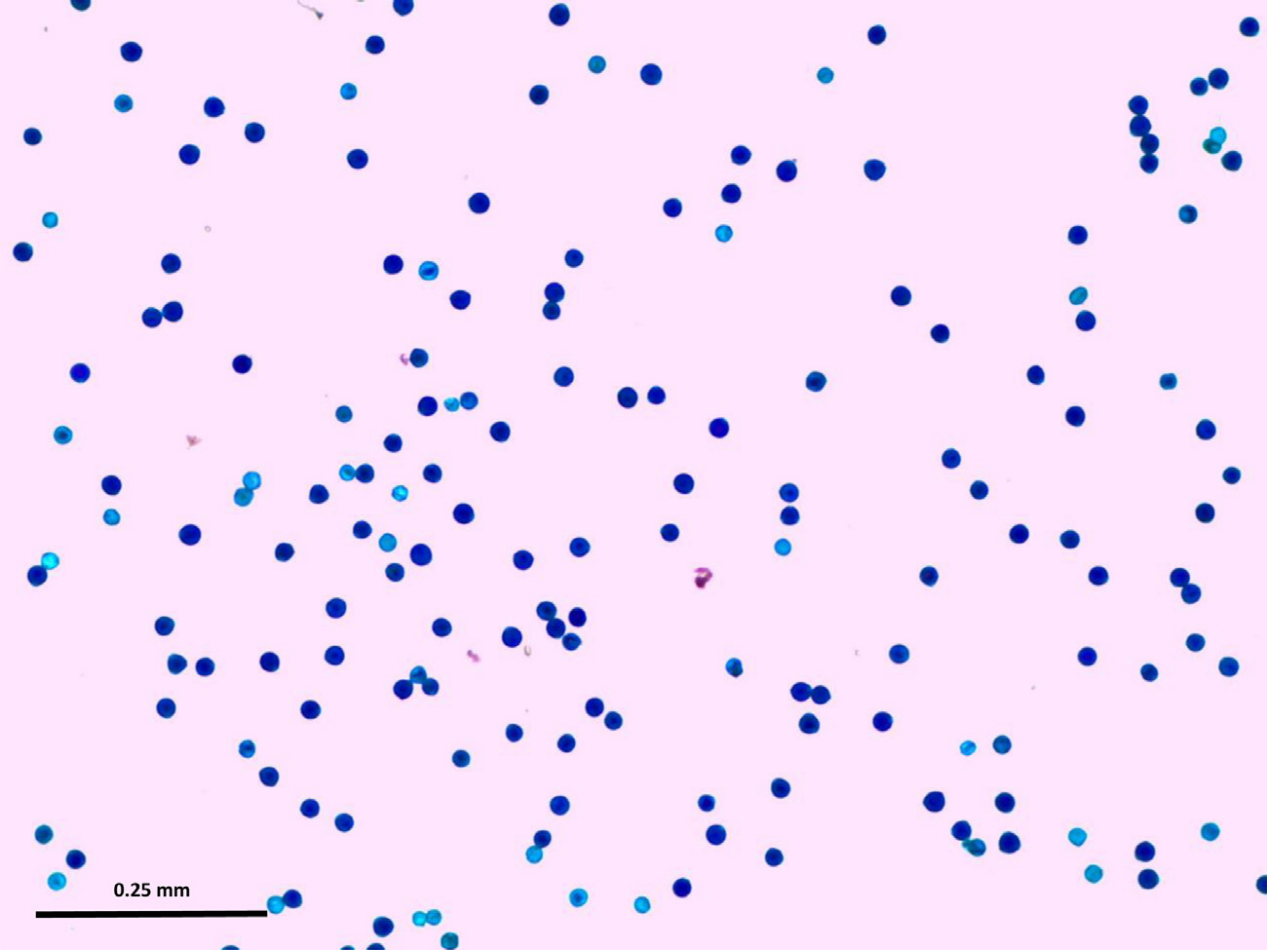
Image with good staining intensity for PollenCounter.

- PollenCounter requires high quality images for accurate assessment of pollen quality. We recommend that the operator pipettes gently while loading the solution containing pollen grains on the slides. This will avoid addition of excess foreign matter which negatively affects accuracy;
- Leave the pollen in the staining solution for about 4 h to allow better staining. Pollen grain on images acquired right after the pollen is released into the staining solution are poorly stained;
- Thoroughly vortex the staining solution containing the pollen to enhance release and avoid clumping of pollen grains. If the pollen grains are over-clustered, the “watershed” algorithm fails to separate them which results in underestimation of the number of pollen grains (total number and viable pollen) compromising the accuracy of the estimate;
- Estimate difference in pollen size and circularity between viable and total pollen to appropriately define the range of these parameters to include in the macro;
- PollenCounter is based on color recognition as the number of pollen on red and green channels depends on pollen initial color. It is necessary to adjust the contrast [13], brightness of the camera while acquiring the images. When the contrast is low and viable pollen grains are not sufficiently dark blue, the total number of pollen will be underestimated and the number of viable pollen would be overestimated. The contrast can also be adjusted in ImageJ using the command [run(“Enhance Contrast…”, “saturated=xx”)] with xx the percentage of statured pixels to achieve a clear distinction between light blue and dark blue stained pollen. The user should be careful in using this command to avoid over staining of the pollen that could lead to an overestimating number of viable pollen. An example of an image with good staining intensity for PollenCounter is shown in Fig. 4.

*Future improvement*

Assessing pollen thermo-tolerance is important to identify thermo-tolerant genotypes. However, pollen can be viable but fail to germinate, which negatively affects fruits set. For this purpose, the percentage of germinated pollen could be a better indicator of thermo-tolerance. The current specifications of PollenCounter are not adapted to automatically assess the percentage of germinated pollen. Further improvement of the PollenCounter or development of other image-based phenotyping techniques to assess the percentage of pollen germination would improve the efficiency of the identification of new sources of thermo-tolerance in crops.

### Method validation: assessment of pollen thermo-tolerance using pollencounter

**Fig. 5:**
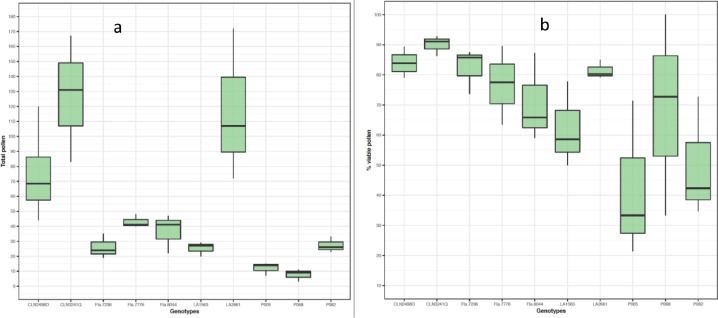
Distribution of pollen number (a) and pollen viability (b) of ten tomato genotypes using PollenCounter.

We assessed whether PollenCounter can discriminate tomato genotypes based on pollen number and proportion of pollen viability under moderate heat stress conditions (see Additional information). For this purpose, we selected the Scenario 1, which showed the highest correlation with manual counting for both pollen number and proportion of pollen viability. The Macro with Scenario 1 was applied to microscopic pollen grains images of the 10 genotypes. Our results showed that PollenCounter was able to distinguish genotypes with high and low total pollen, and high and low proportion of viable pollen. The accession LA2661 (‘Nagcarlang’) is well-known for its high pollen production and pollen viability [1] and this is confirmed by our assessment using PollenCounter (Fig. 5a,b). Similarly, CLN3241Q and CLN2498D were developed by the World Vegetable Center and were reported to possess fair and moderate fruit set under heat stress, respectively (https://avrdc.org/seed/improved-lines/fresh-market-tomato/). In sum, we have optimized the PollenCounter to accurately measure pollen number and viability as well as distinguish the response to heat stress among different tomato genotypes.

### Plant growing environment

Ten non-improved and improved tomato (*Solanum lycopersicum* L.) genotypes (Table S1) were raised in a greenhouse using standard agronomic practices. The average relative humidity and daily temperatures during the plants’ growth cycle ranged from 77.5 ± 14.1% (day time) to 97 ± 3% (night time) and 26 ± 1°C (night time) to 30 ± 3°C (day time), respectively. These conditions were sufficient to create moderate heat stress on the tomato plants and negatively impair pollen development (Peet et al. [6].

**Table S1:** Pollen donors for the optimization of PollenCounter.

Varieties	Status	Origin
Fla.7236 Fla.8044 Fla.7776	Improved Improved Improved	Florida Agricultural Experiment Station (FAES), University of Florida
P082	Improved	Crop Research Institute, Ghana
P005	Improved	
P068	Improved	
CLN3241Q	Improved	World Vegetable Center
CLN2498D	Improved	
LA2661 LA1563	Non-improved Non-improved	C.M. Rick Tomato Genetic Resources Center, University of California, Davis

## Declaration of Competing Interest

The authors declare that they have no known competing financial interests or personal relationships that could have appeared to influence the work reported in this paper.

## Appendix Supplementary materials

Image, application 1

Image, image 2

Image, image 3

Image, image 4

Image, image 5

Image, image 6

Image, image 7

Image, image 8

Image, image 9

Image, image 10

Image, image 11

Image, image 12

Image, image 13

Image, image 14

Image, image 15

Image, image 16

Image, image 17

Image, image 18

Image, image 19

Image, image 20

Image, image 21

Image, image 22

Image, image 23

Image, image 24

Image, image 25

Image, image 26

Image, image 27

Image, image 28

Image, image 29

Image, image 30

Image, image 31

Image, image 32

Image, application 33

Image, application 34

Image, application 35

Image, application 36

Image, application 37

Image, application 38
