# Supplementary figures and images for: Optimizing pollencounter for high throughput phenotyping of pollen quality in tomatoes
Source: MethodsX. 2020 Jun 27;7:100977. doi: 10.1016/j.mex.2020.100977 (PMC7338780; doi:10.1016/j.mex.2020.100977)

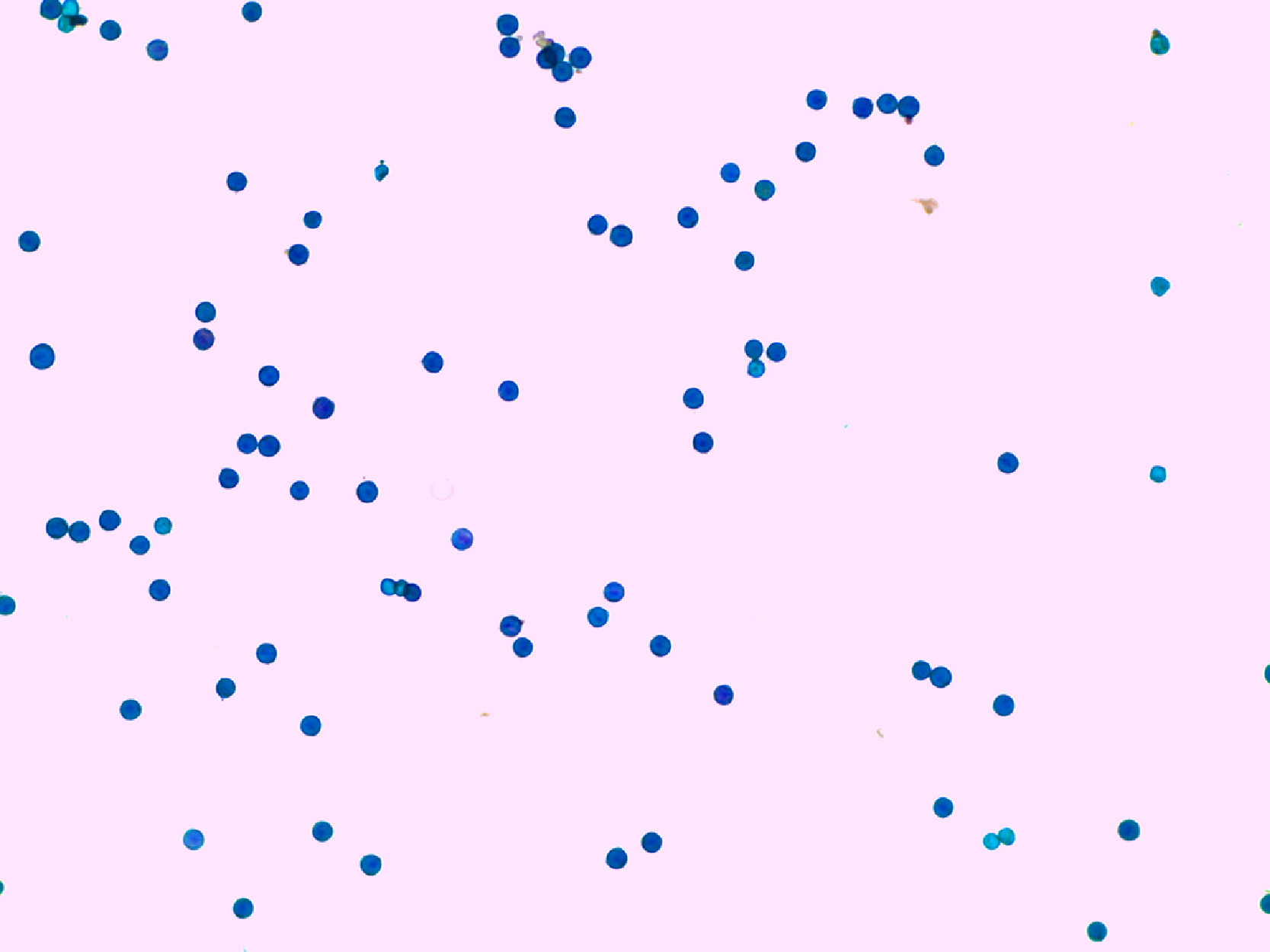

Supplement: Supplementary file 1 [file mmc1.jpg]

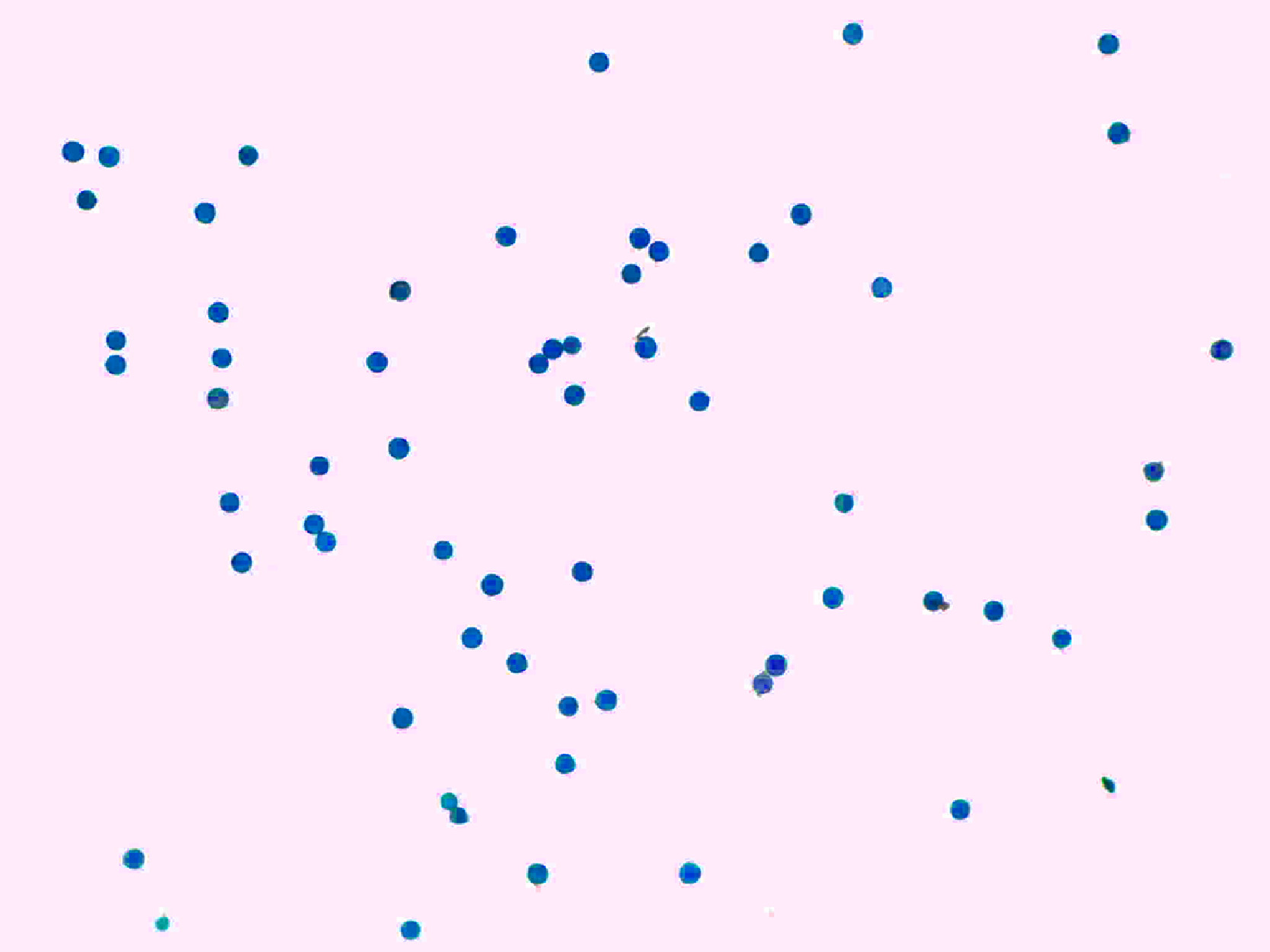

Supplement: Supplementary file 2 [file mmc2.jpg]

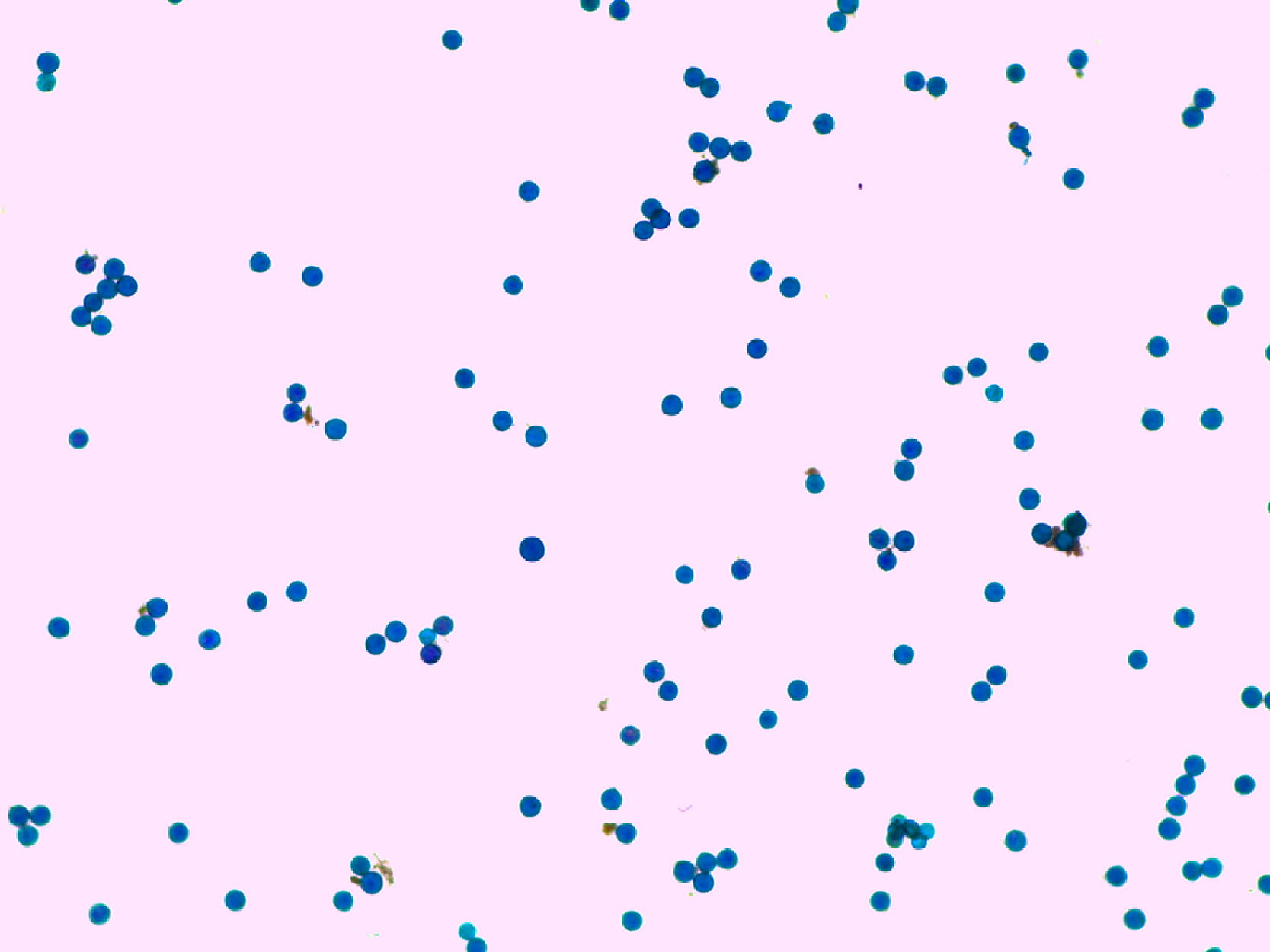

Supplement: Supplementary file 3 [file mmc3.jpg]

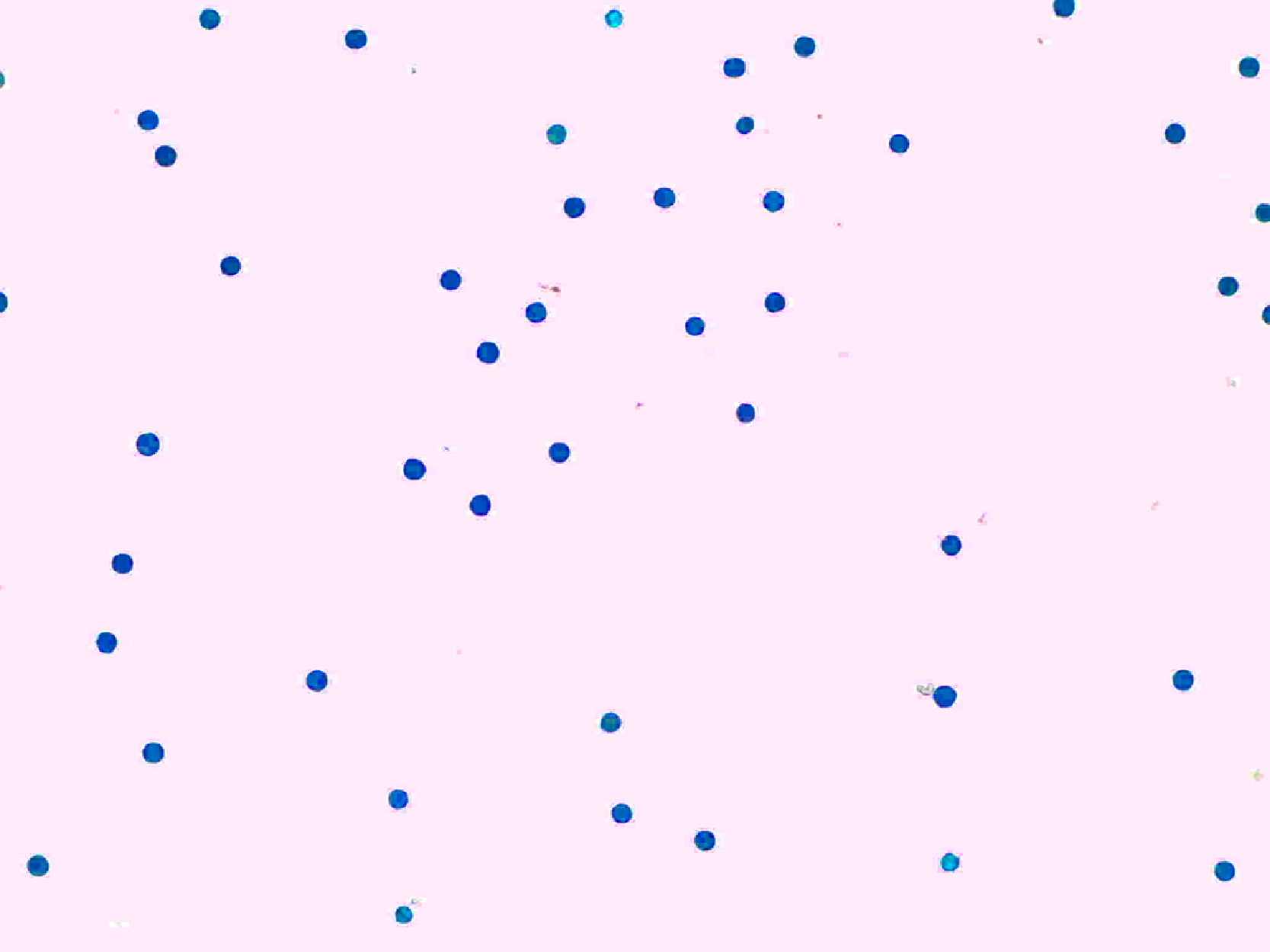

Supplement: Supplementary file 4 [file mmc4.jpg]

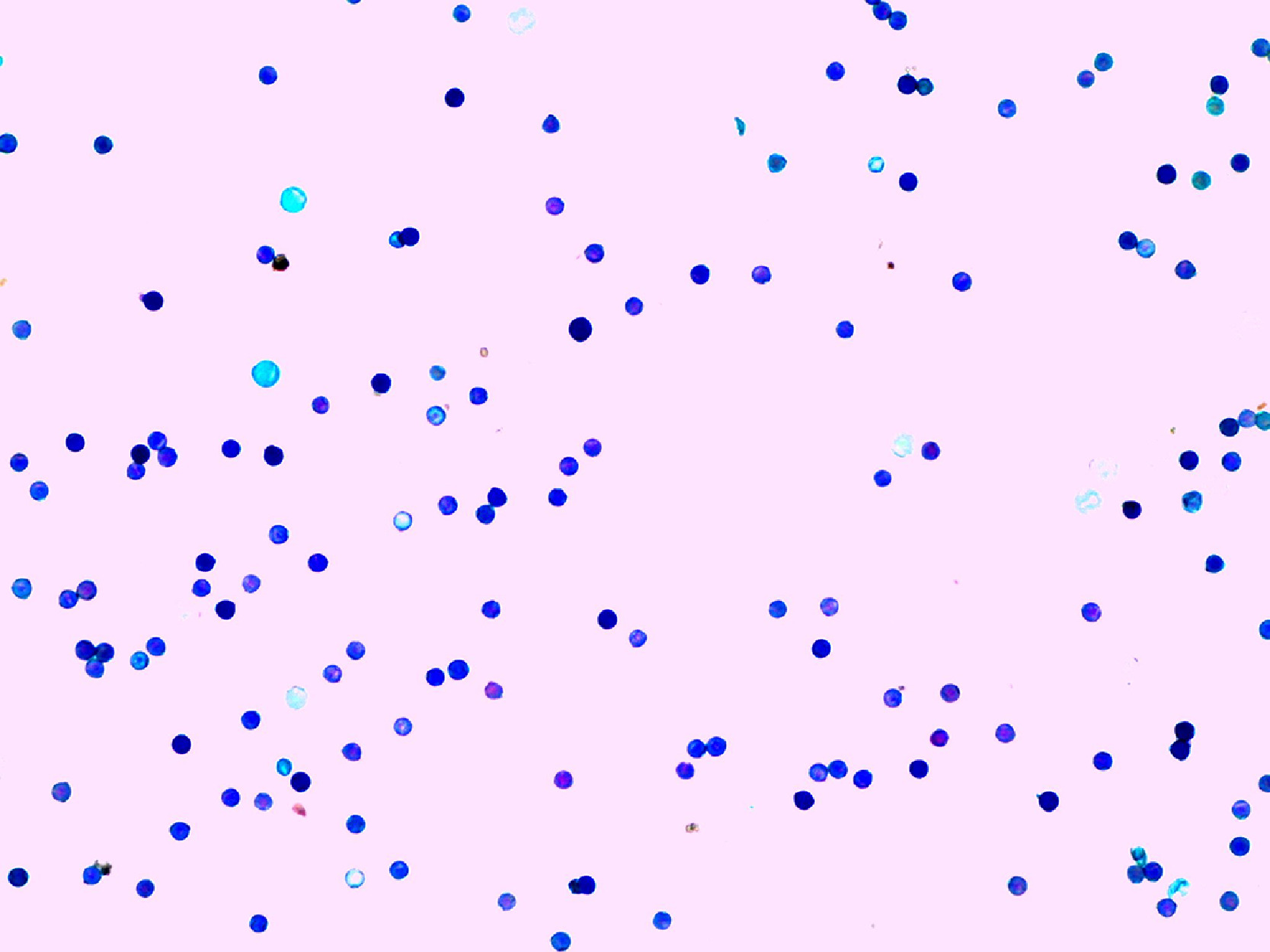

Supplement: Supplementary file 5 [file mmc5.jpg]

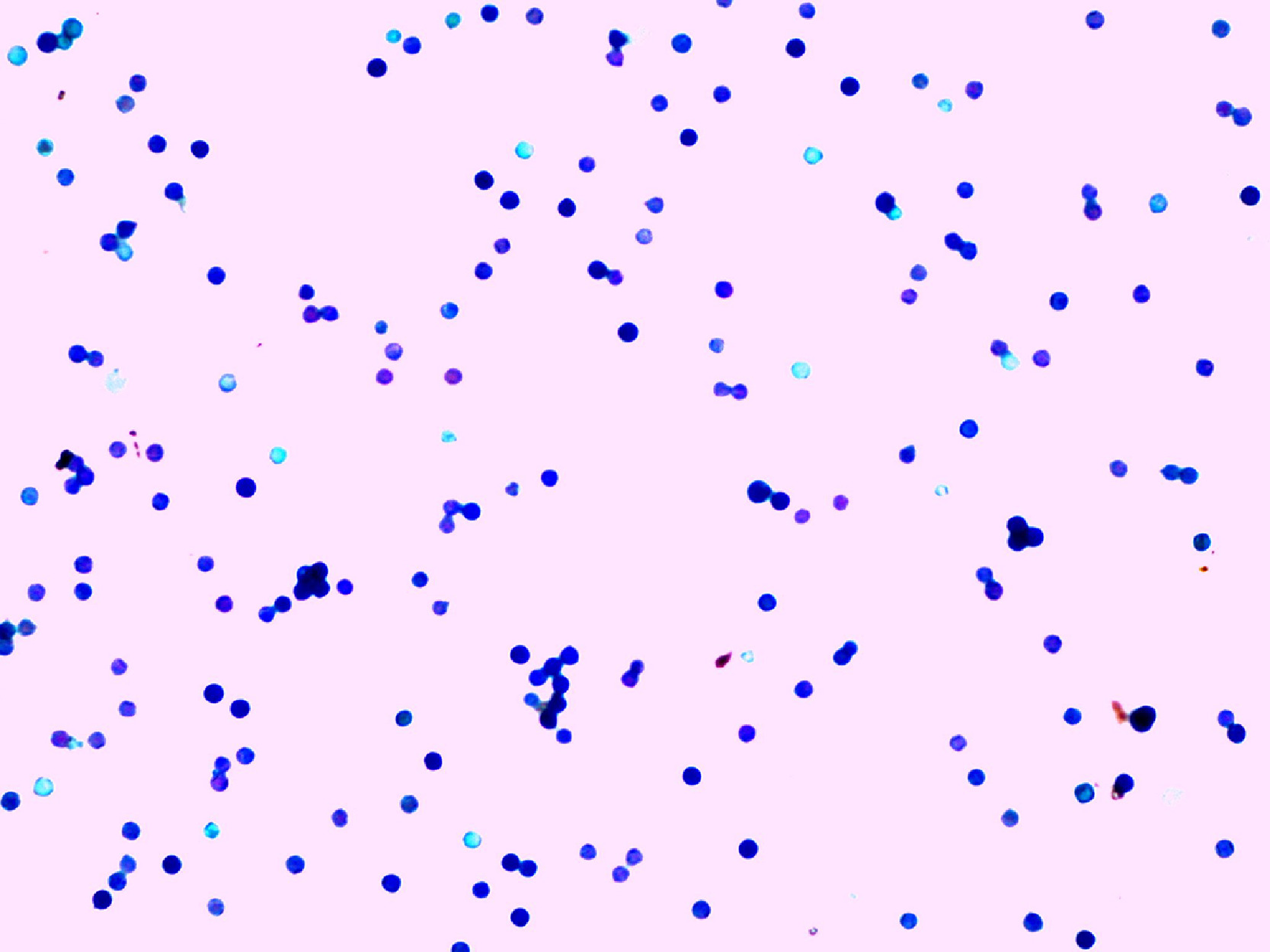

Supplement: Supplementary file 6 [file mmc6.jpg]

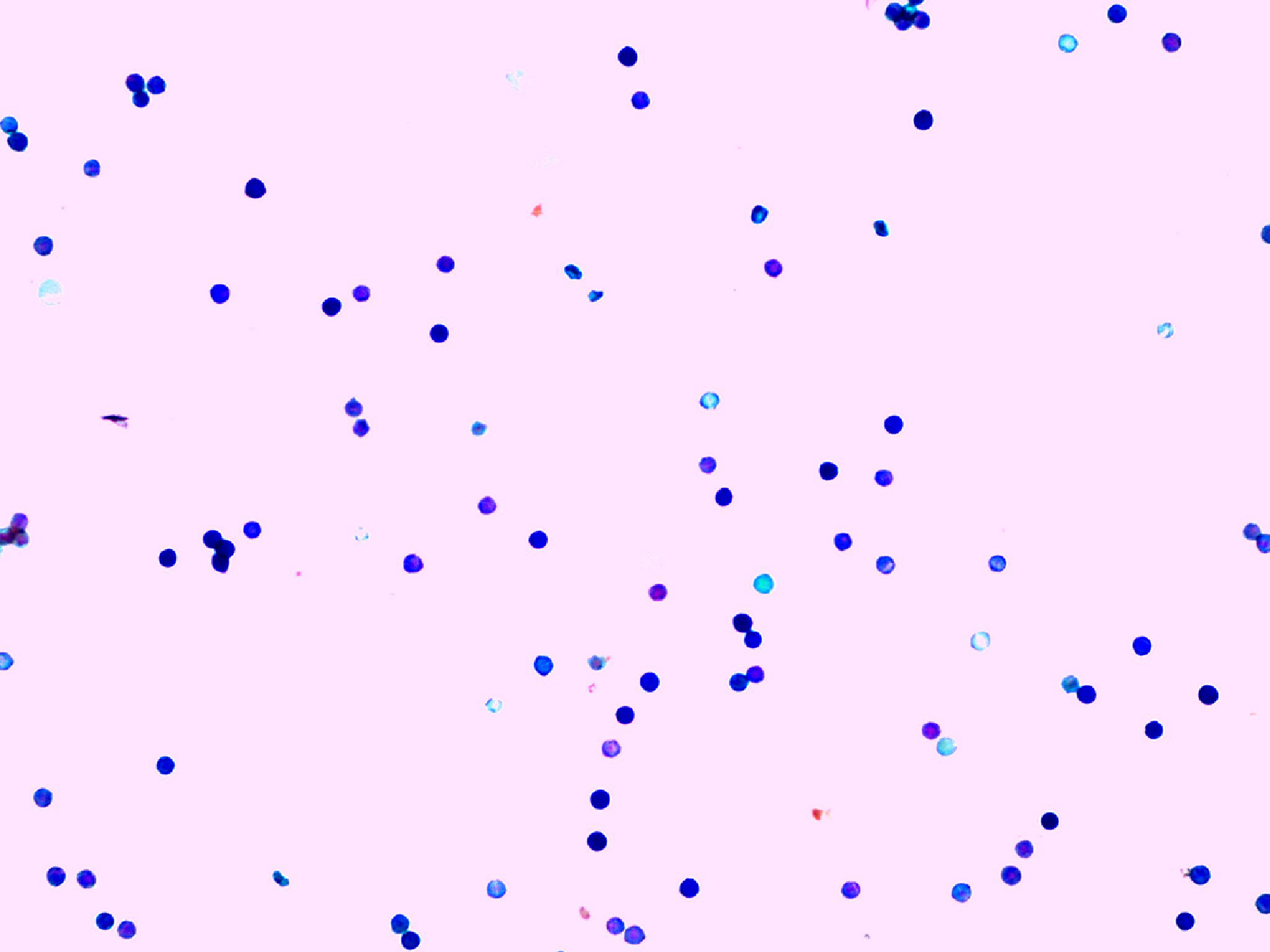

Supplement: Supplementary file 7 [file mmc7.jpg]

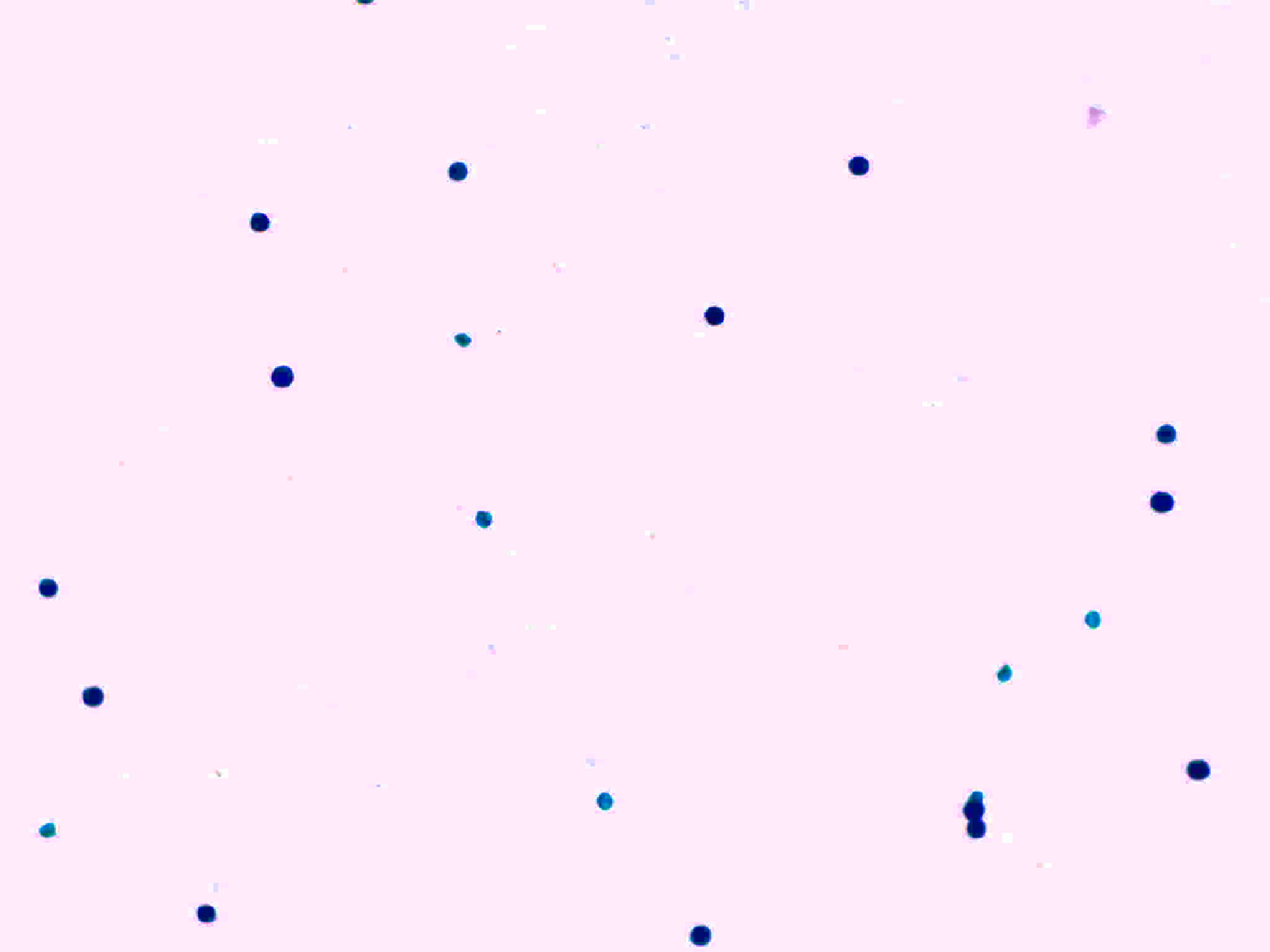

Supplement: Supplementary file 8 [file mmc8.jpg]

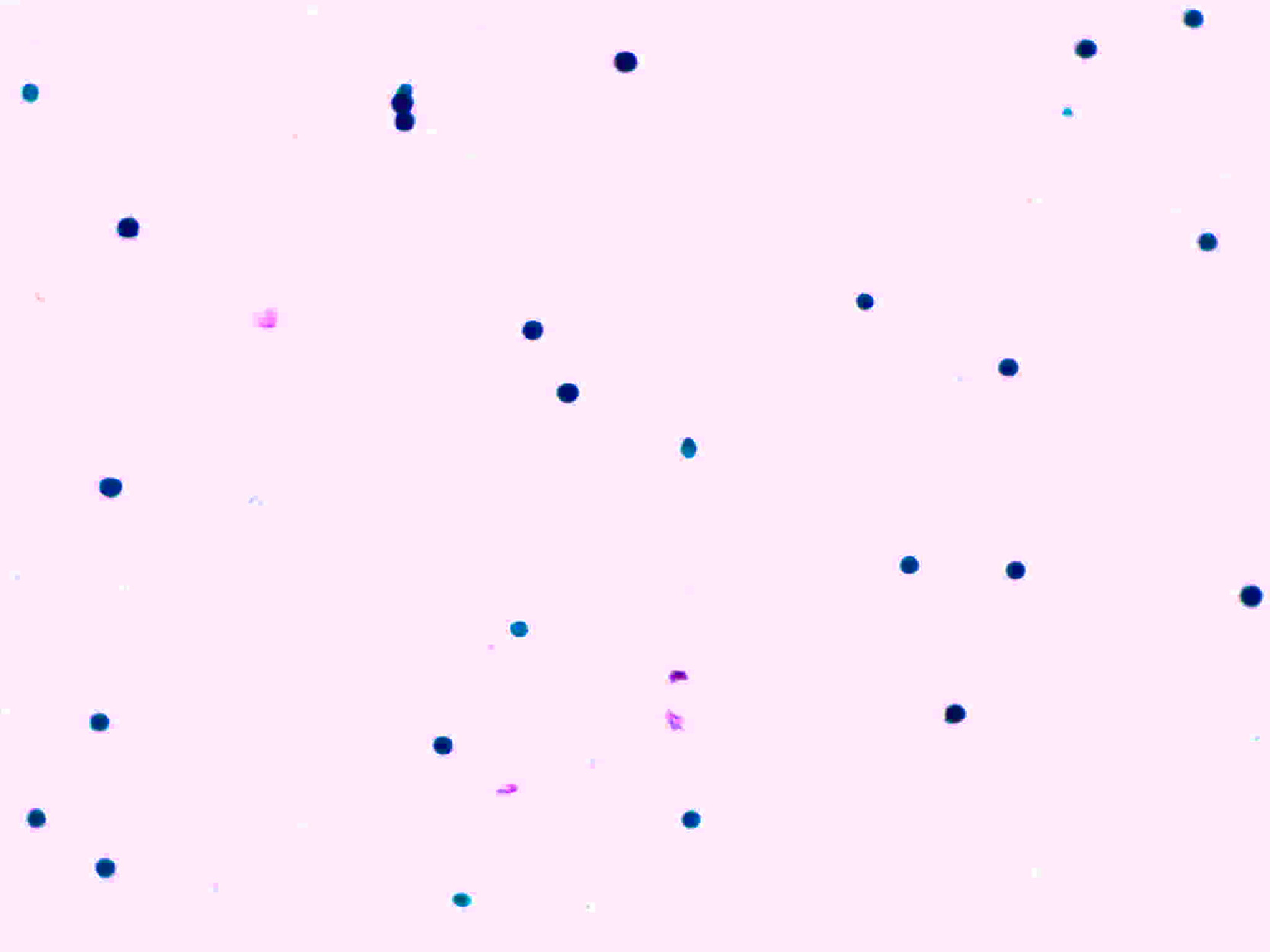

Supplement: Supplementary file 9 [file mmc9.jpg]

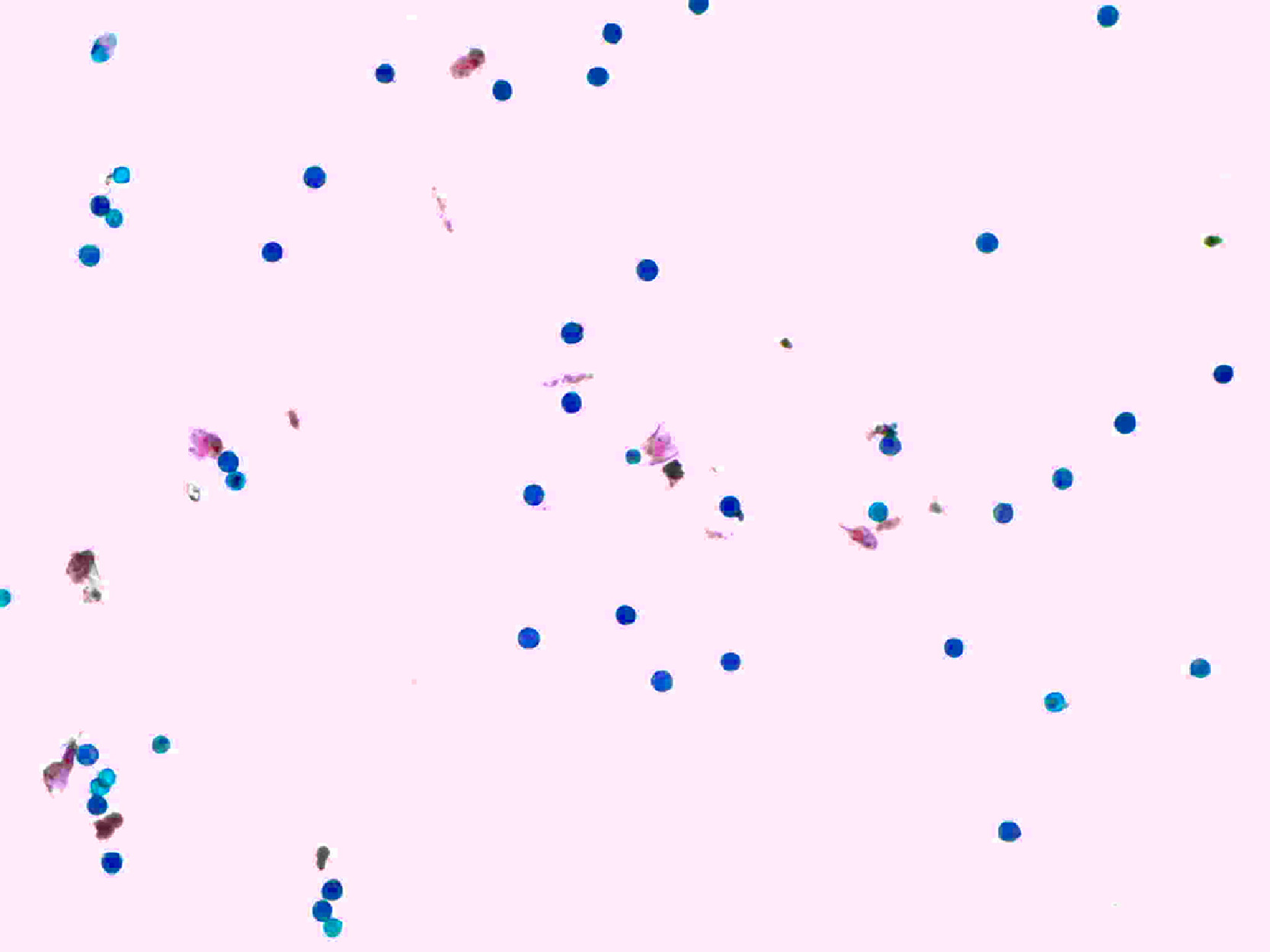

Supplement: Supplementary file 10 [file mmc10.jpg]

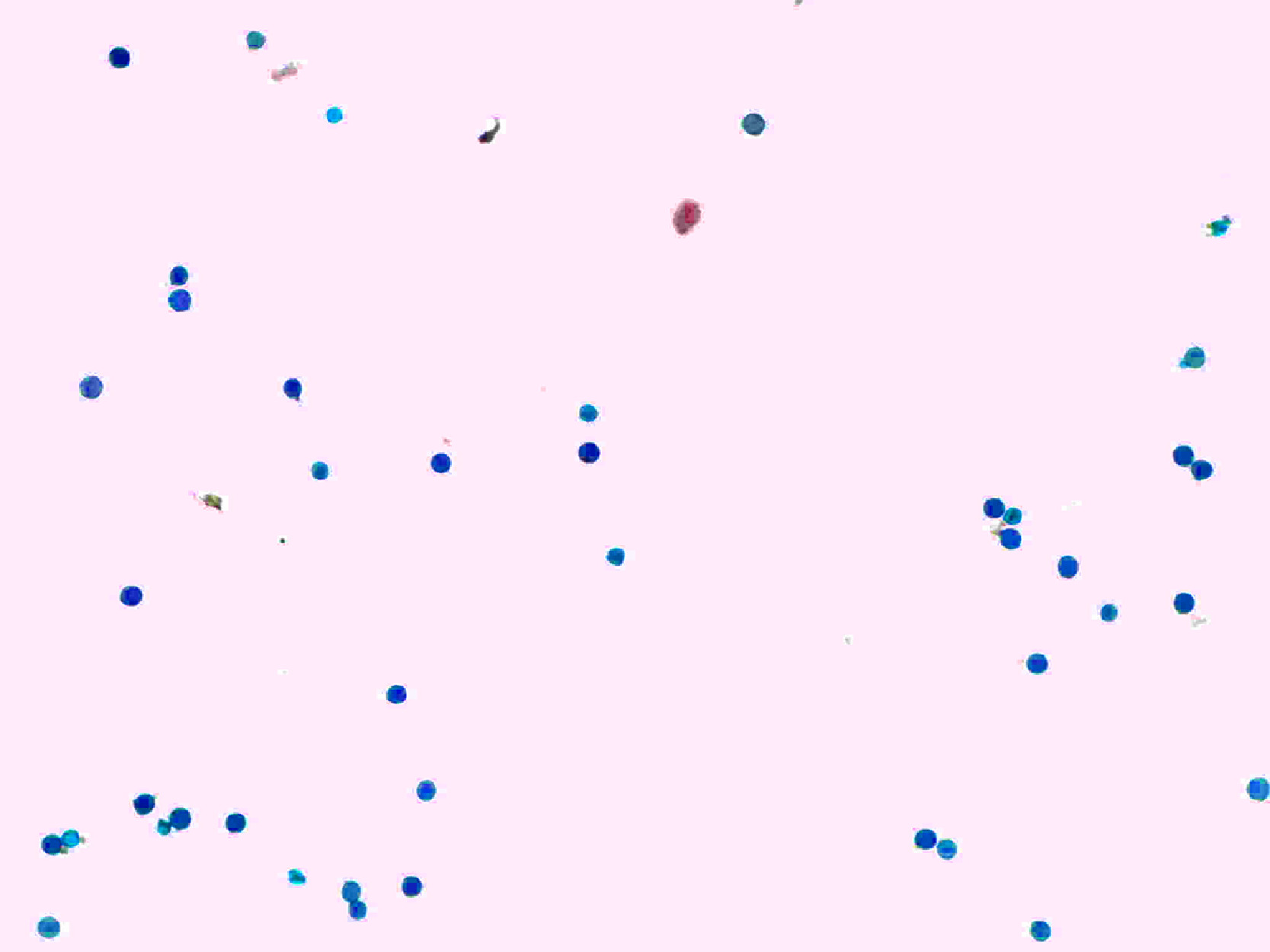

Supplement: Supplementary file 11 [file mmc11.jpg]

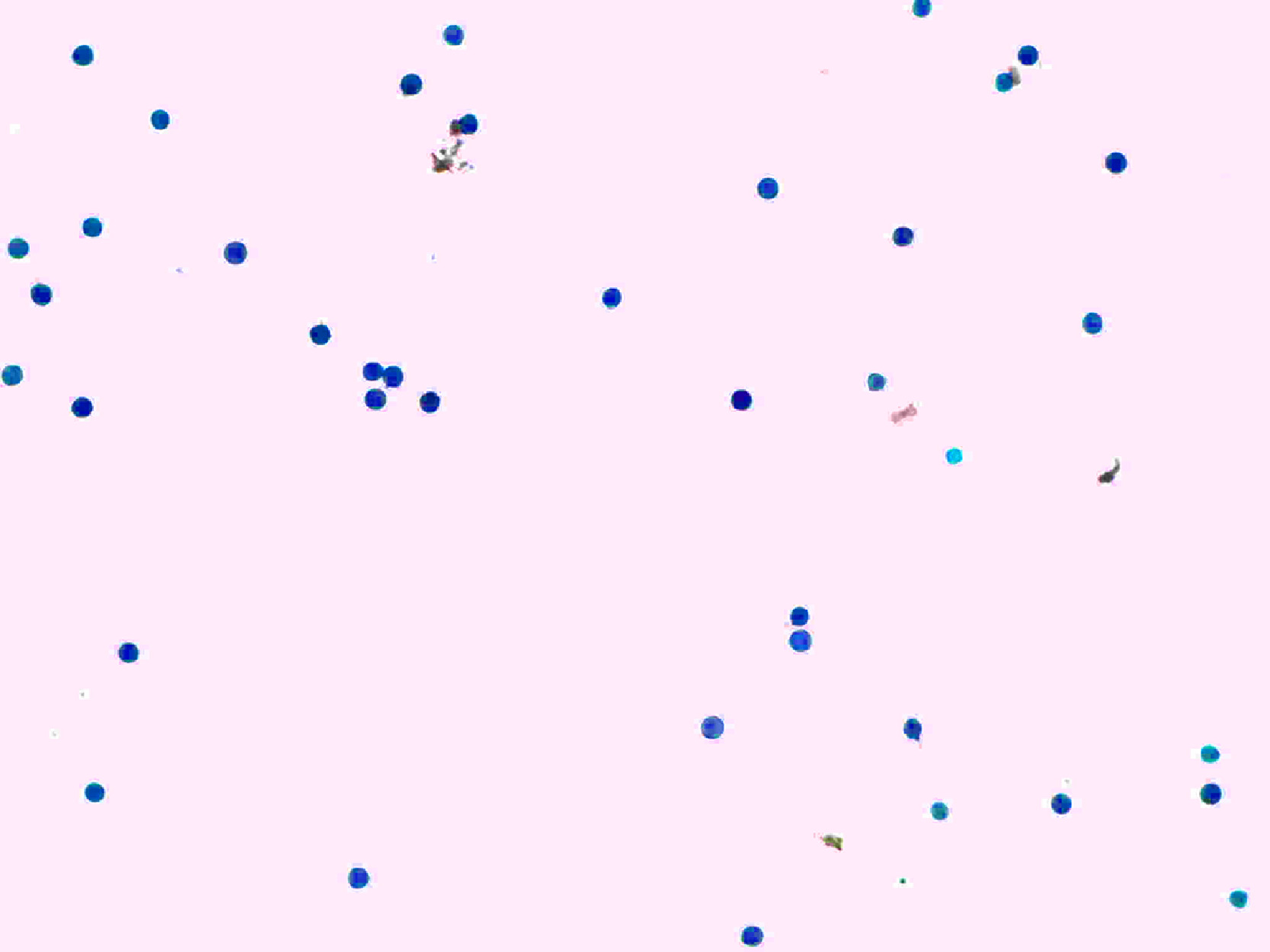

Supplement: Supplementary file 12 [file mmc12.jpg]

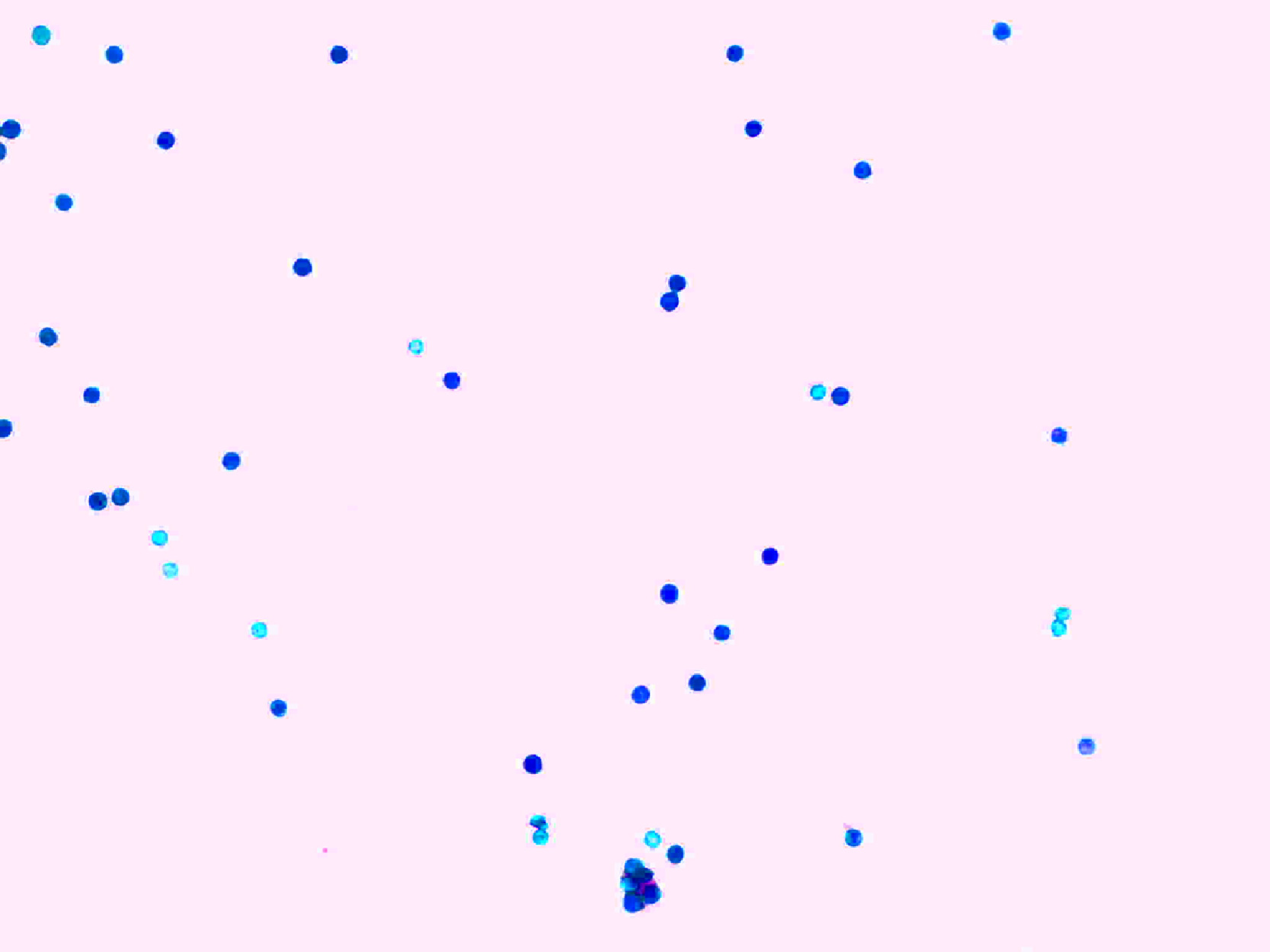

Supplement: Supplementary file 13 [file mmc13.jpg]

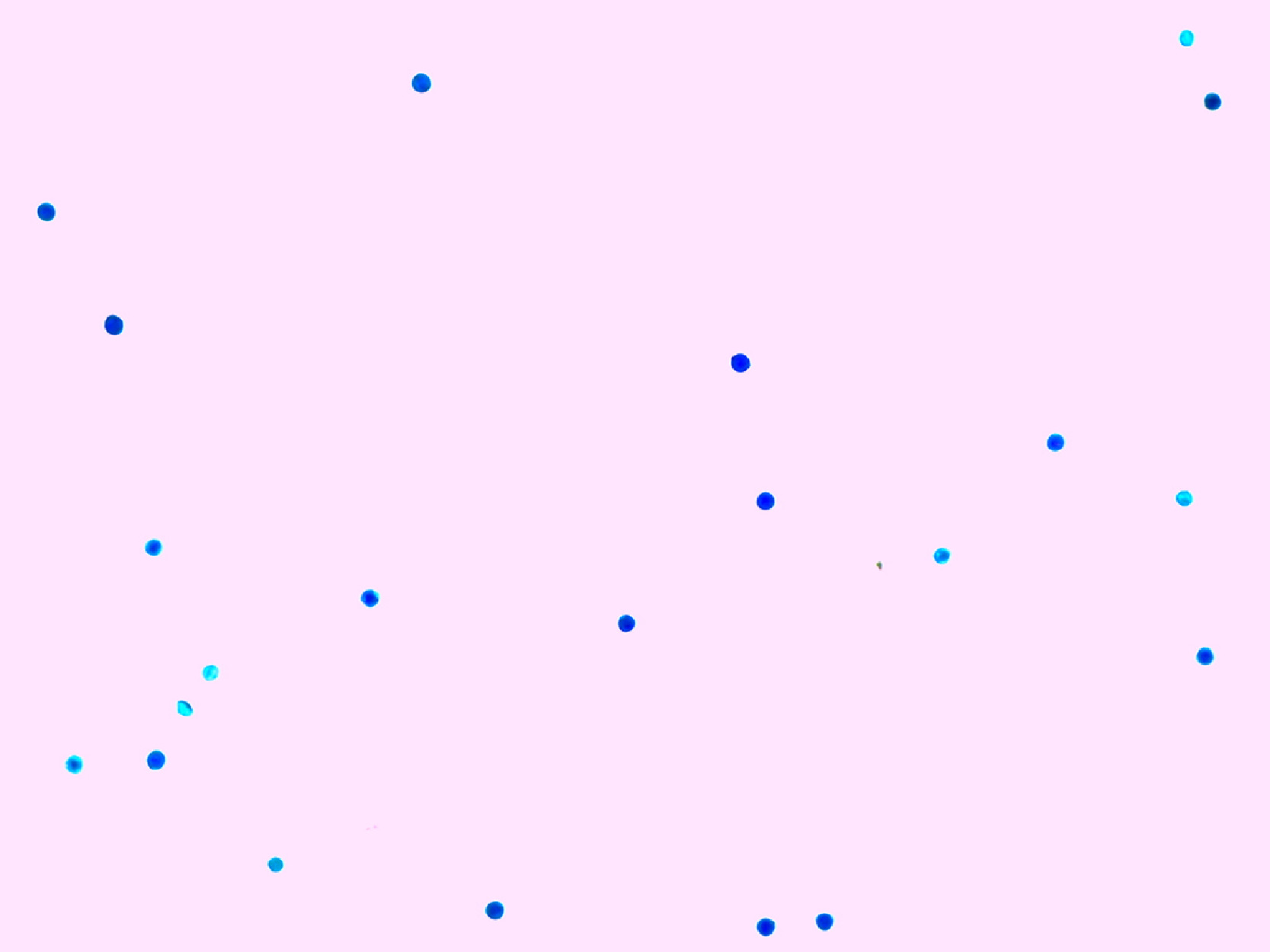

Supplement: Supplementary file 14 [file mmc14.jpg]

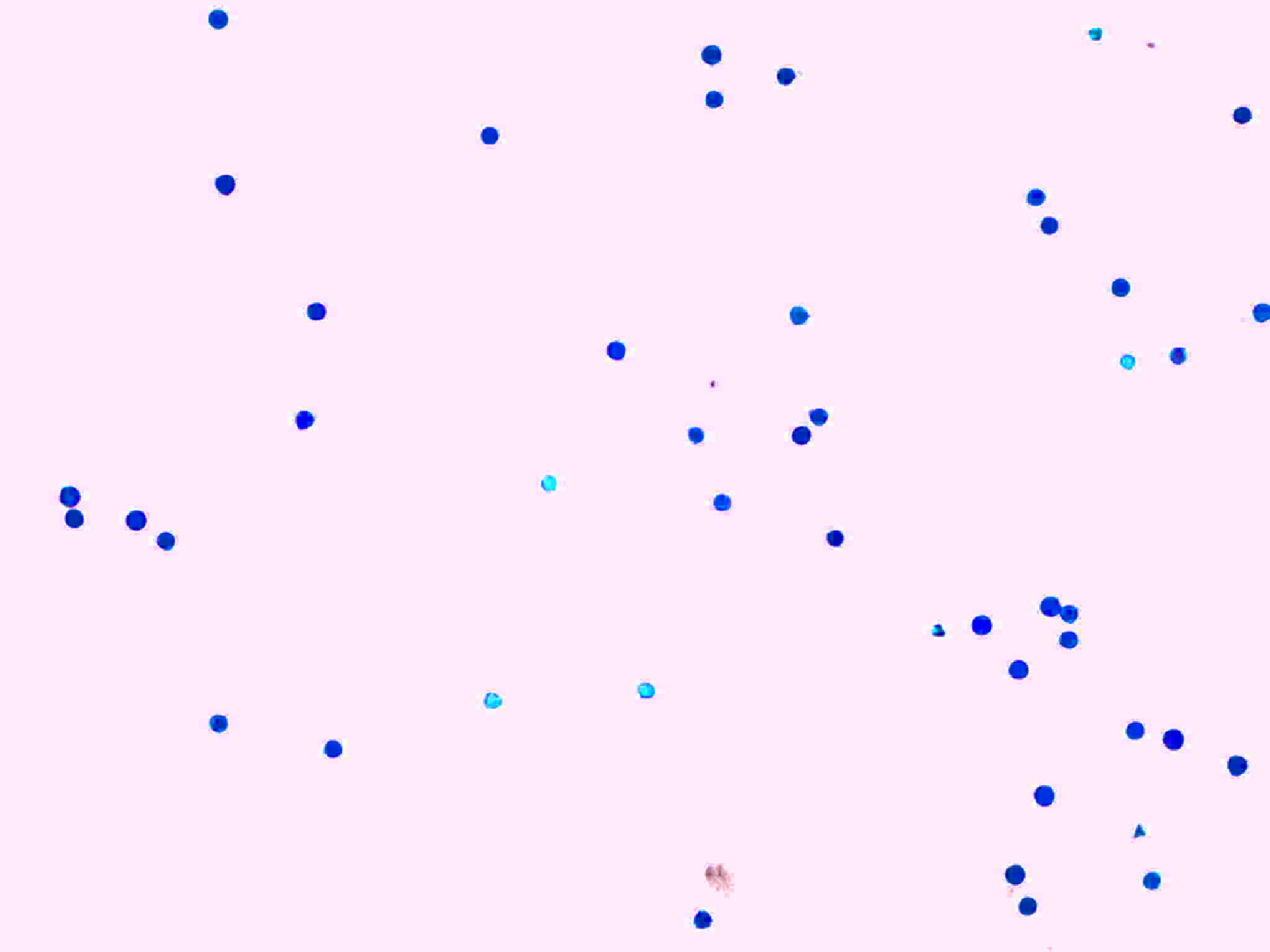

Supplement: Supplementary file 15 [file mmc15.jpg]

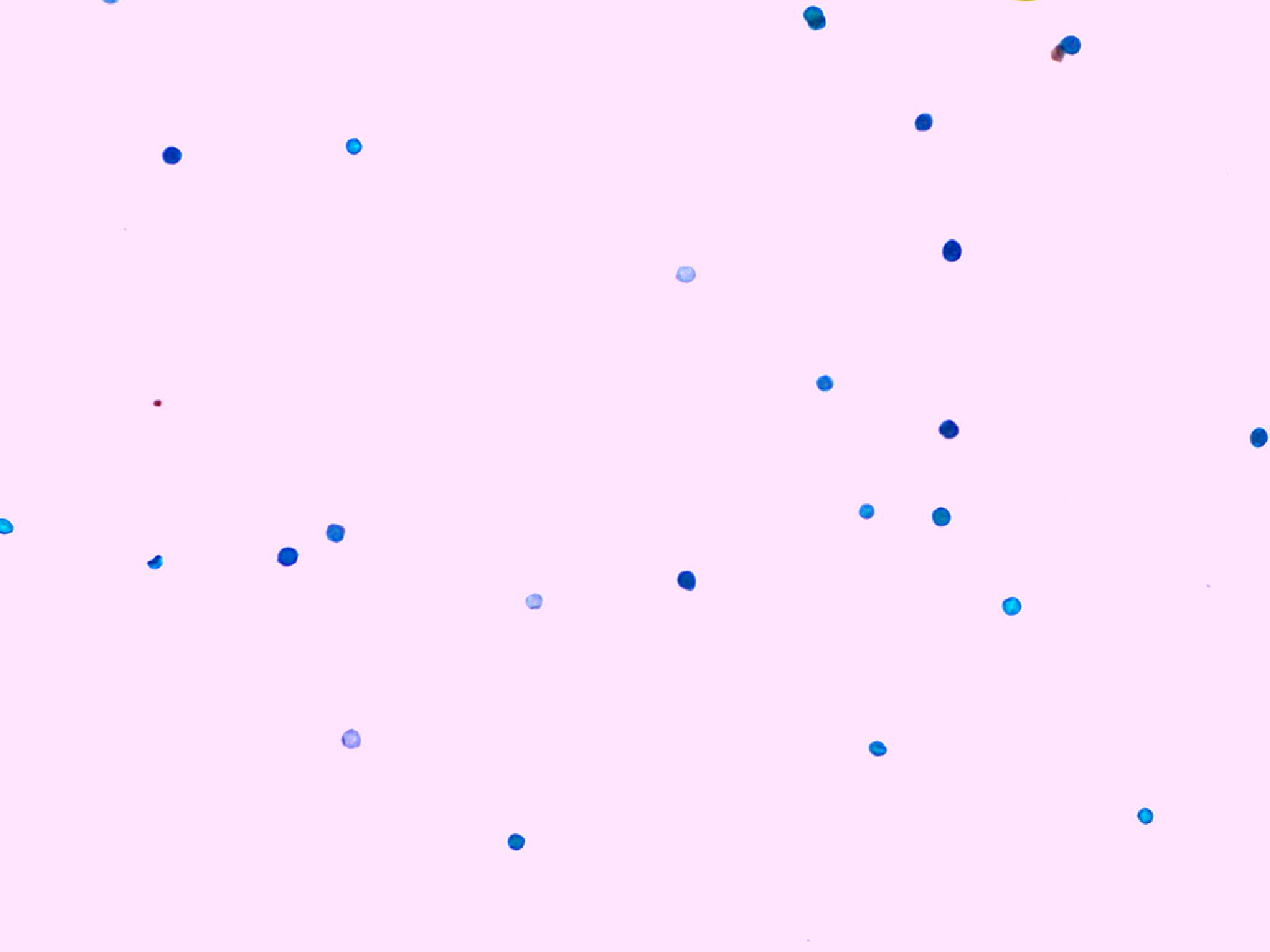

Supplement: Supplementary file 16 [file mmc16.jpg]

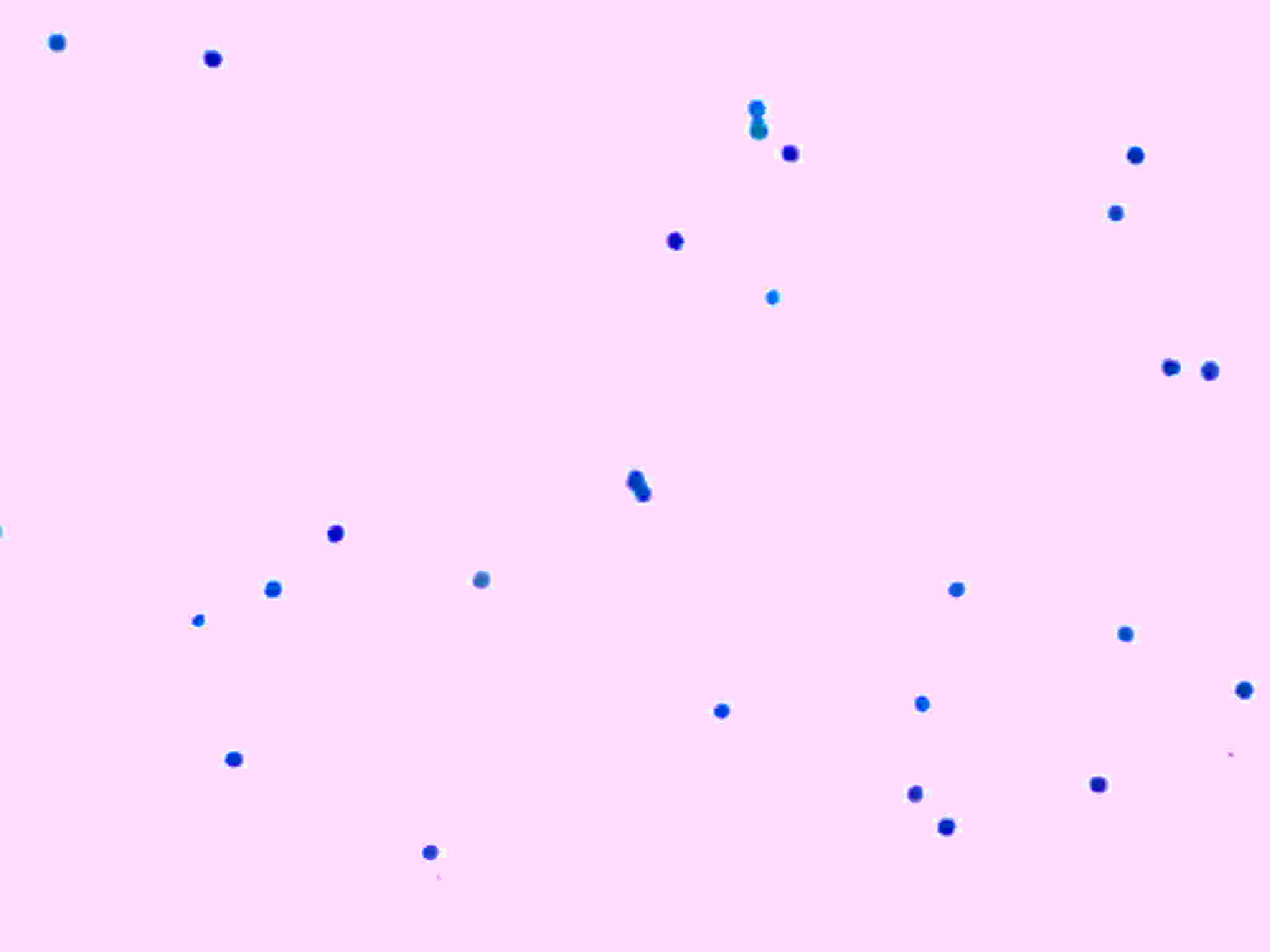

Supplement: Supplementary file 17 [file mmc17.jpg]

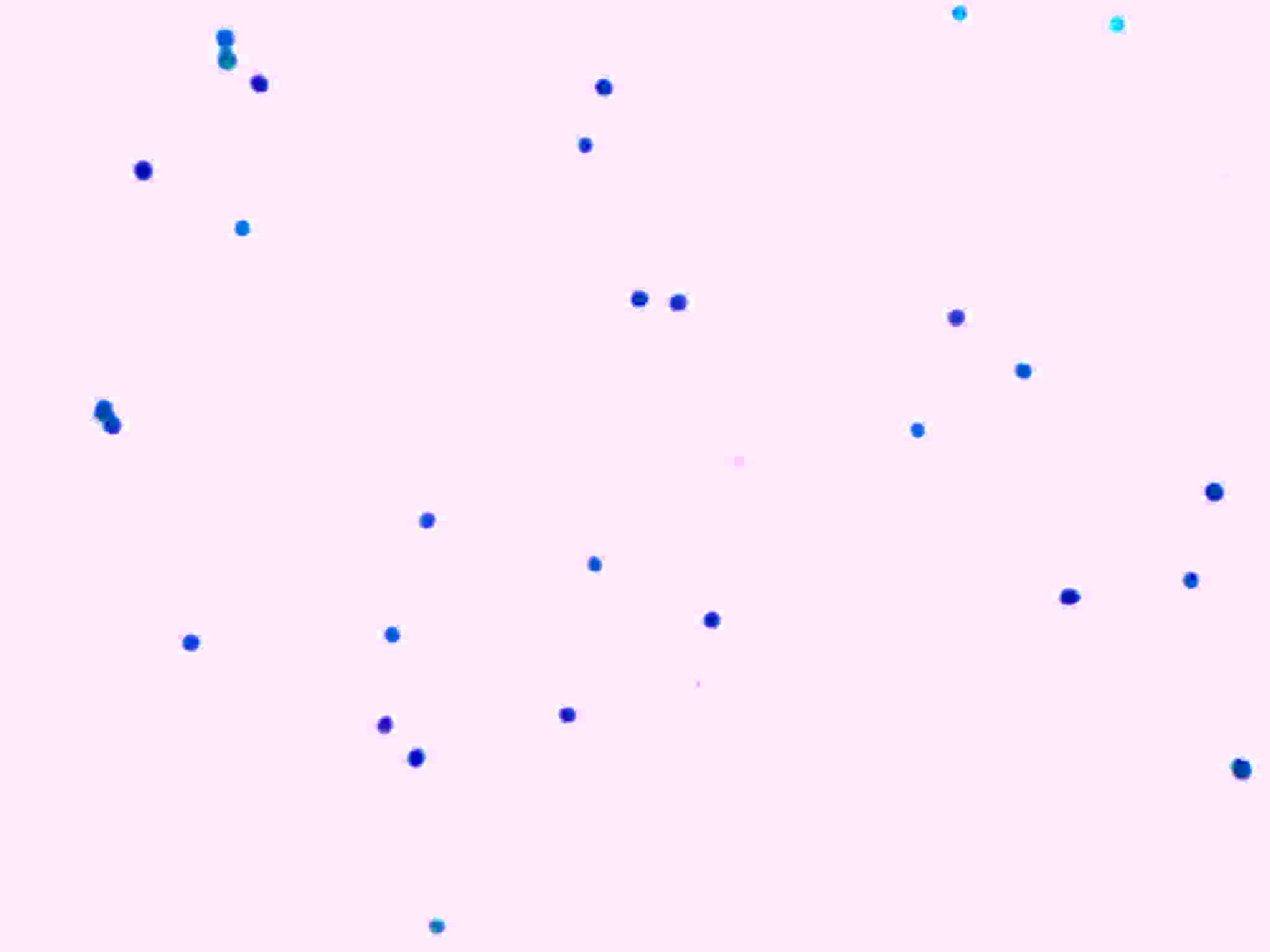

Supplement: Supplementary file 18 [file mmc18.jpg]

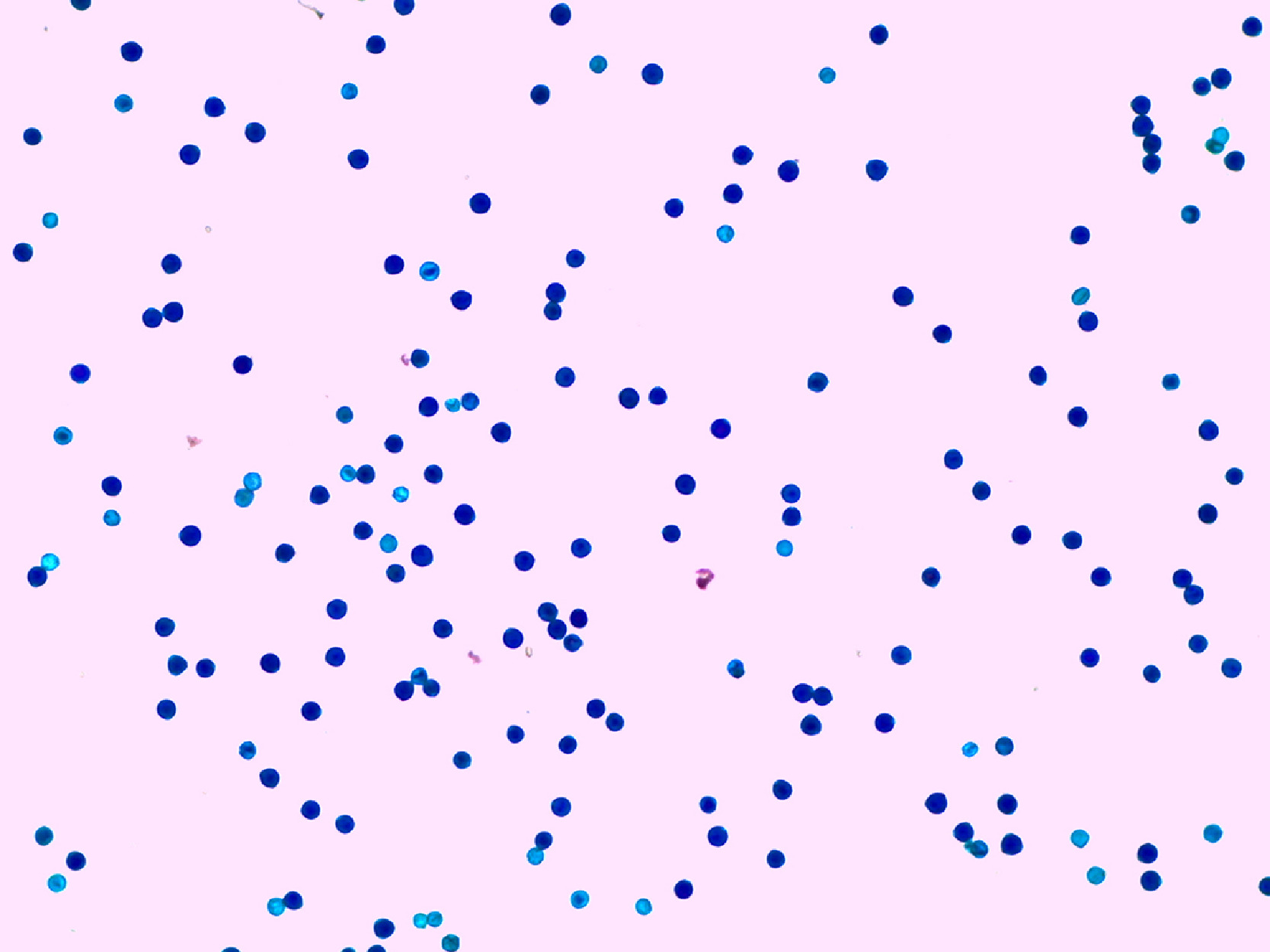

Supplement: Supplementary file 19 [file mmc19.jpg]

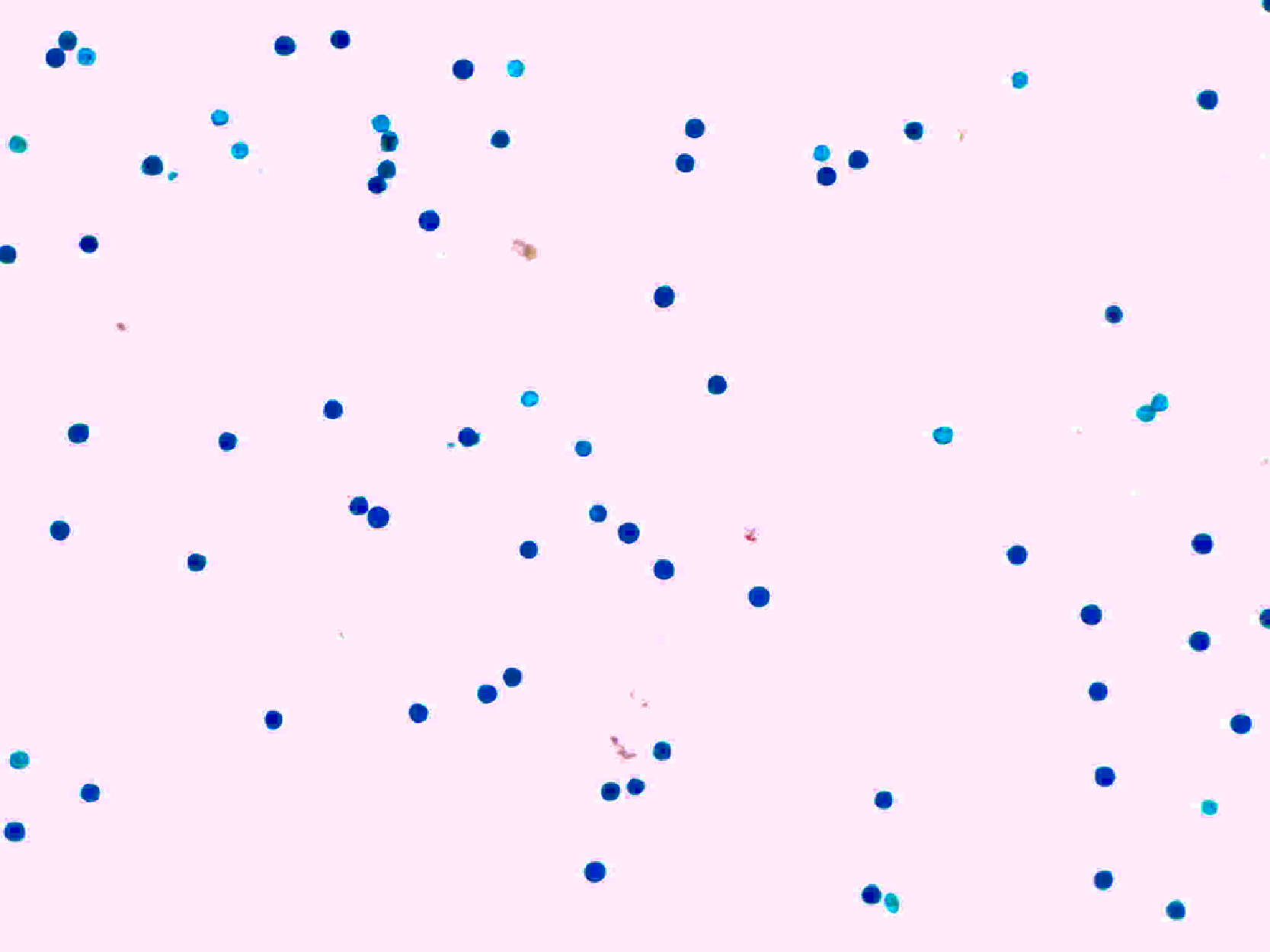

Supplement: Supplementary file 20 [file mmc20.jpg]

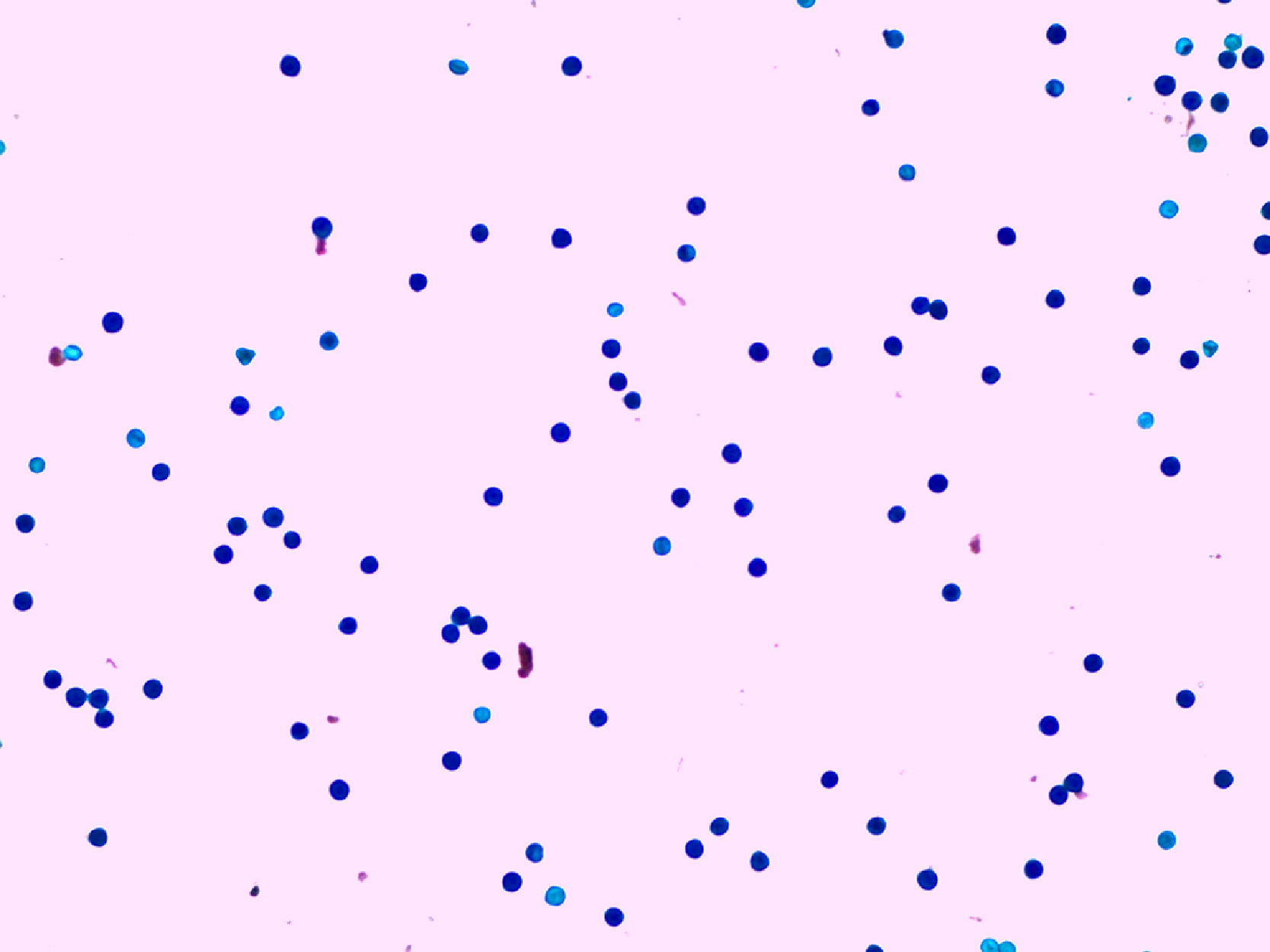

Supplement: Supplementary file 21 [file mmc21.jpg]

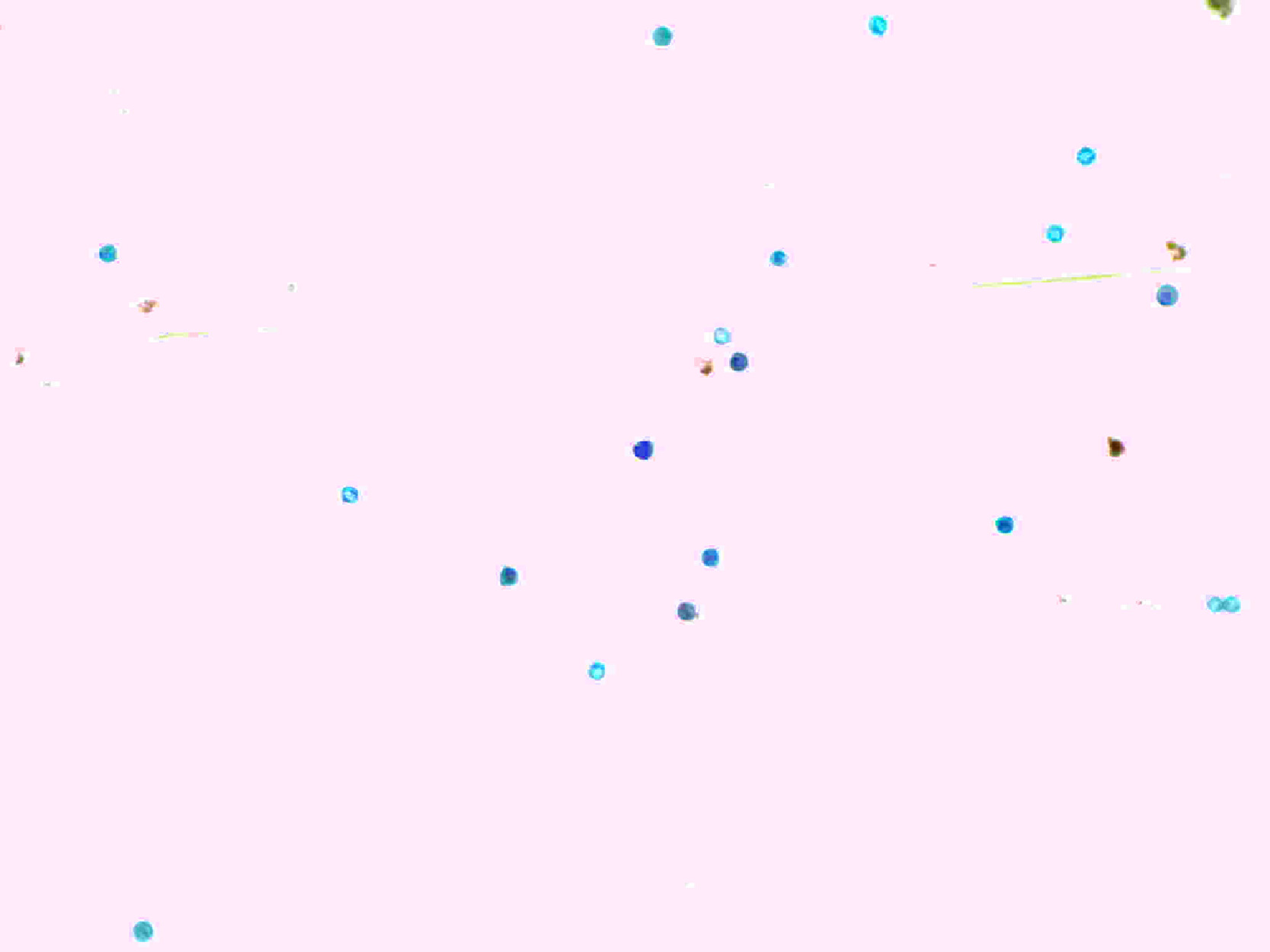

Supplement: Supplementary file 22 [file mmc22.jpg]

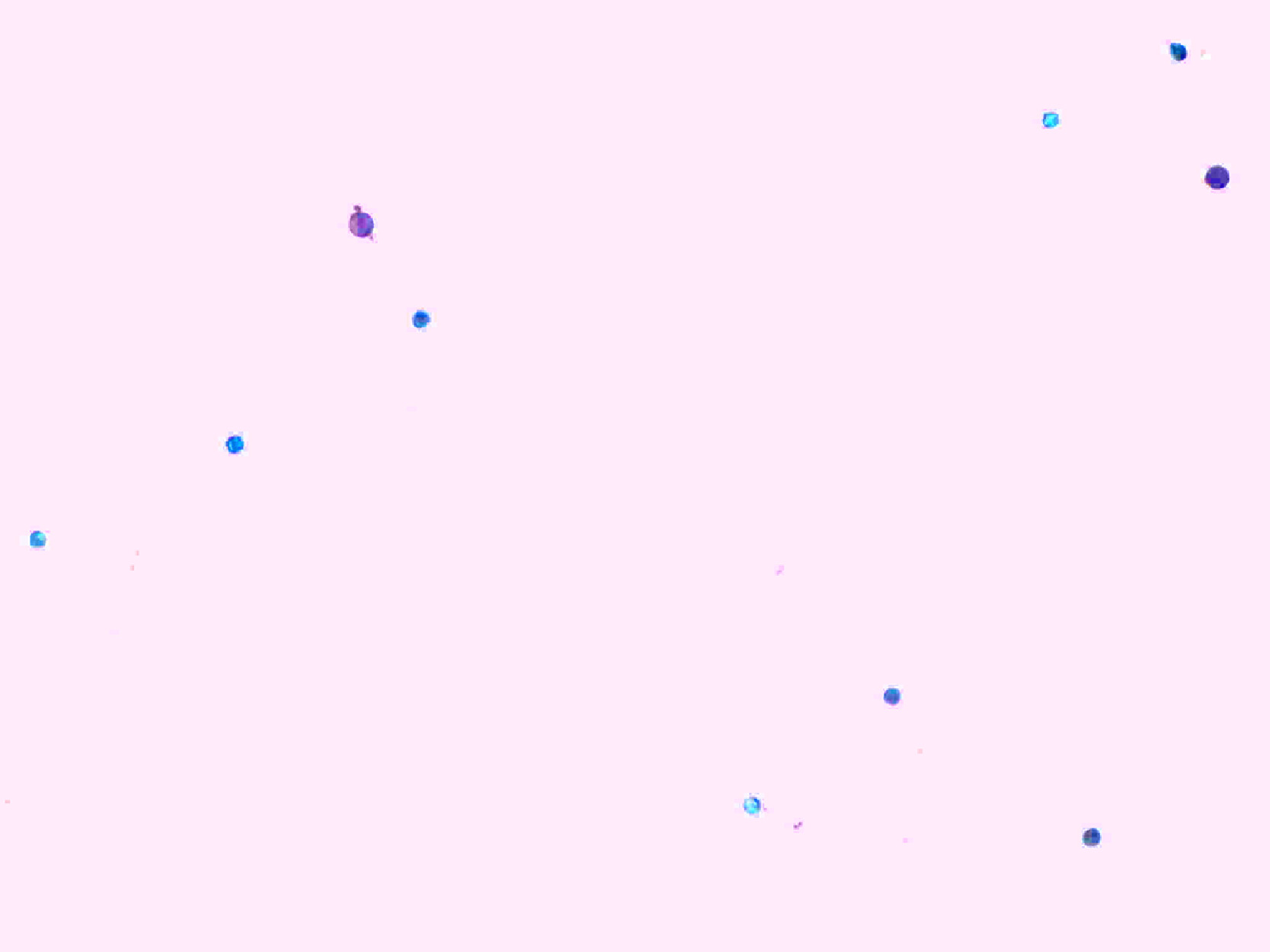

Supplement: Supplementary file 23 [file mmc23.jpg]

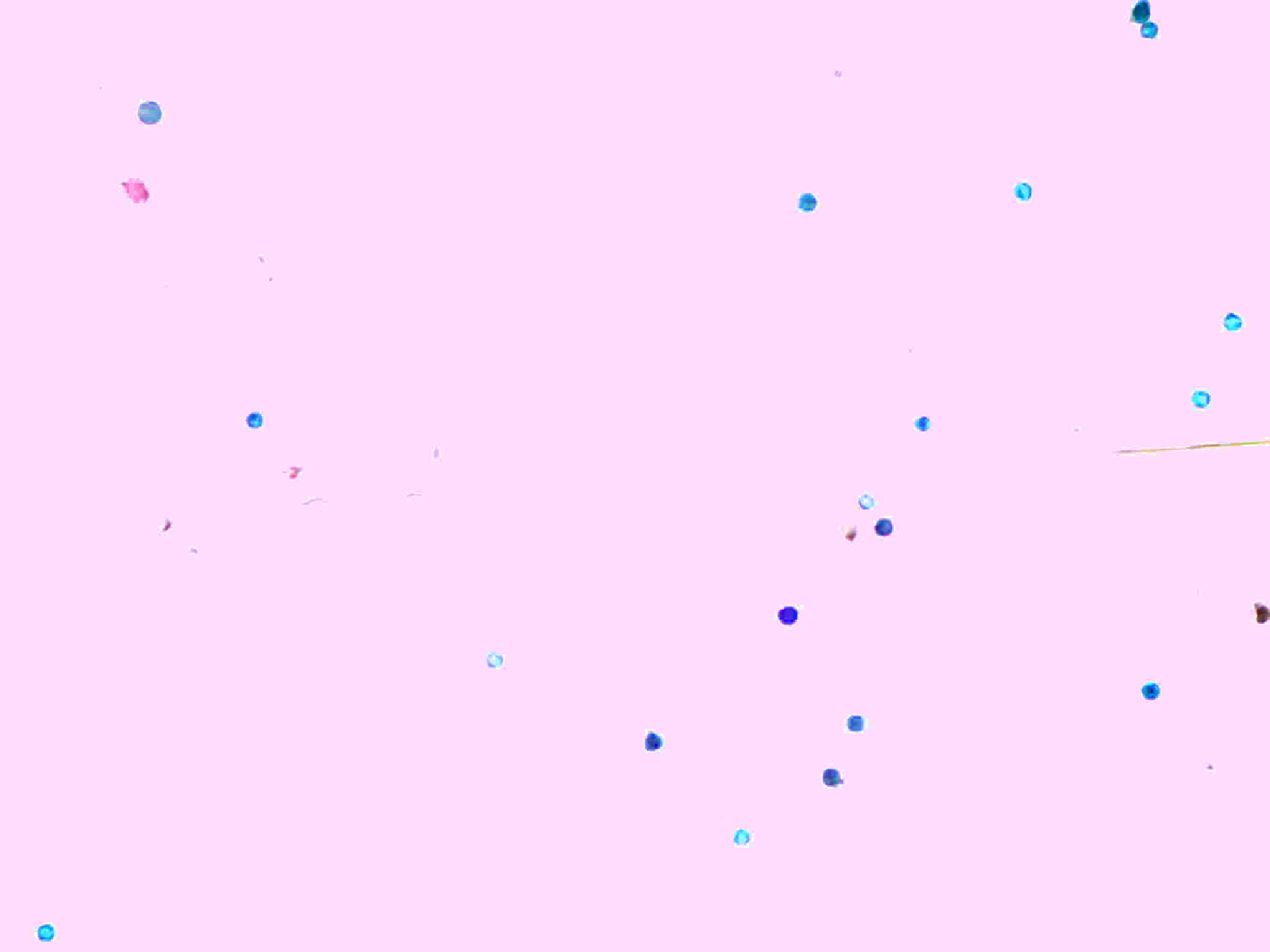

Supplement: Supplementary file 24 [file mmc24.jpg]

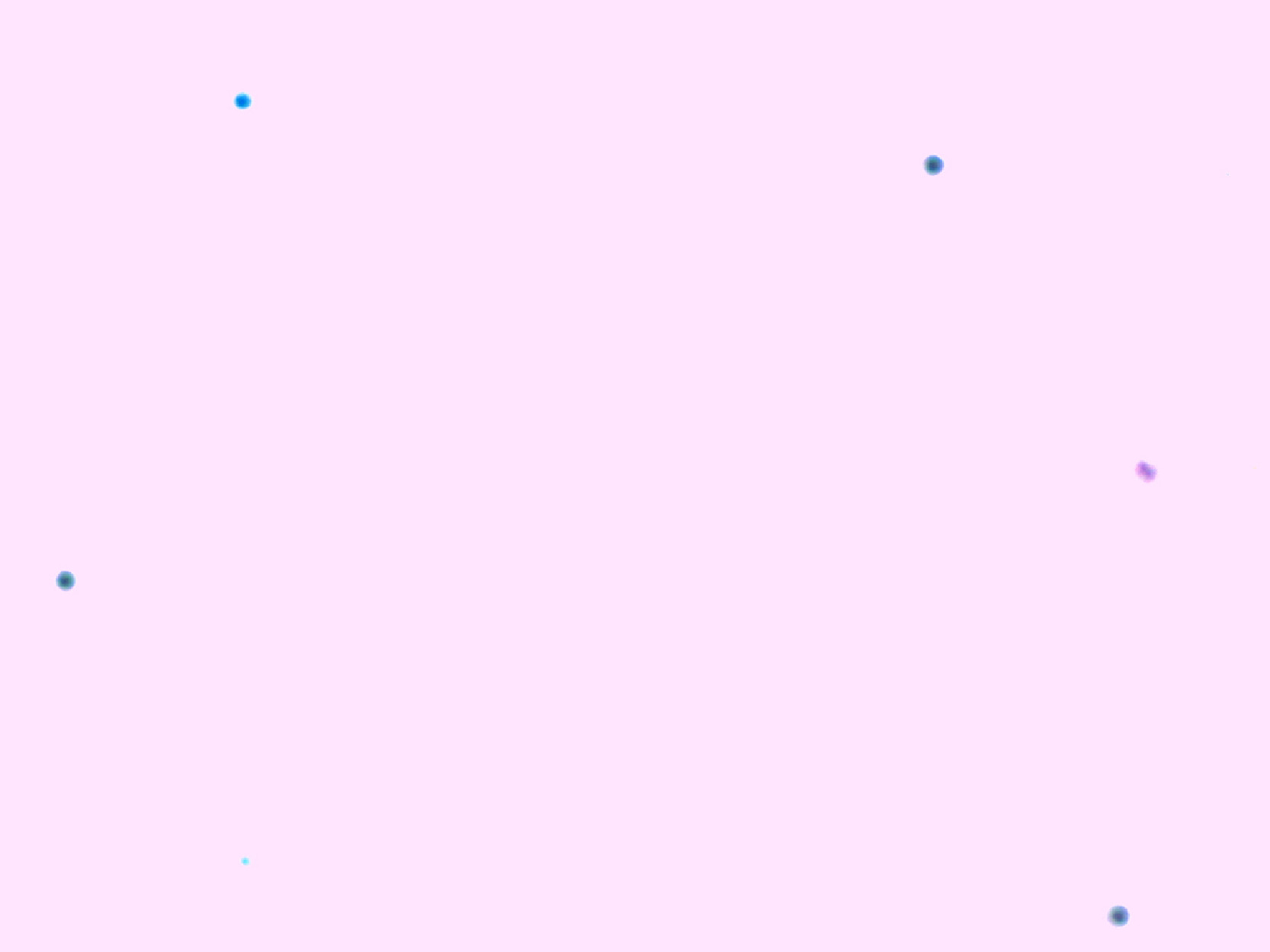

Supplement: Supplementary file 25 [file mmc25.jpg]

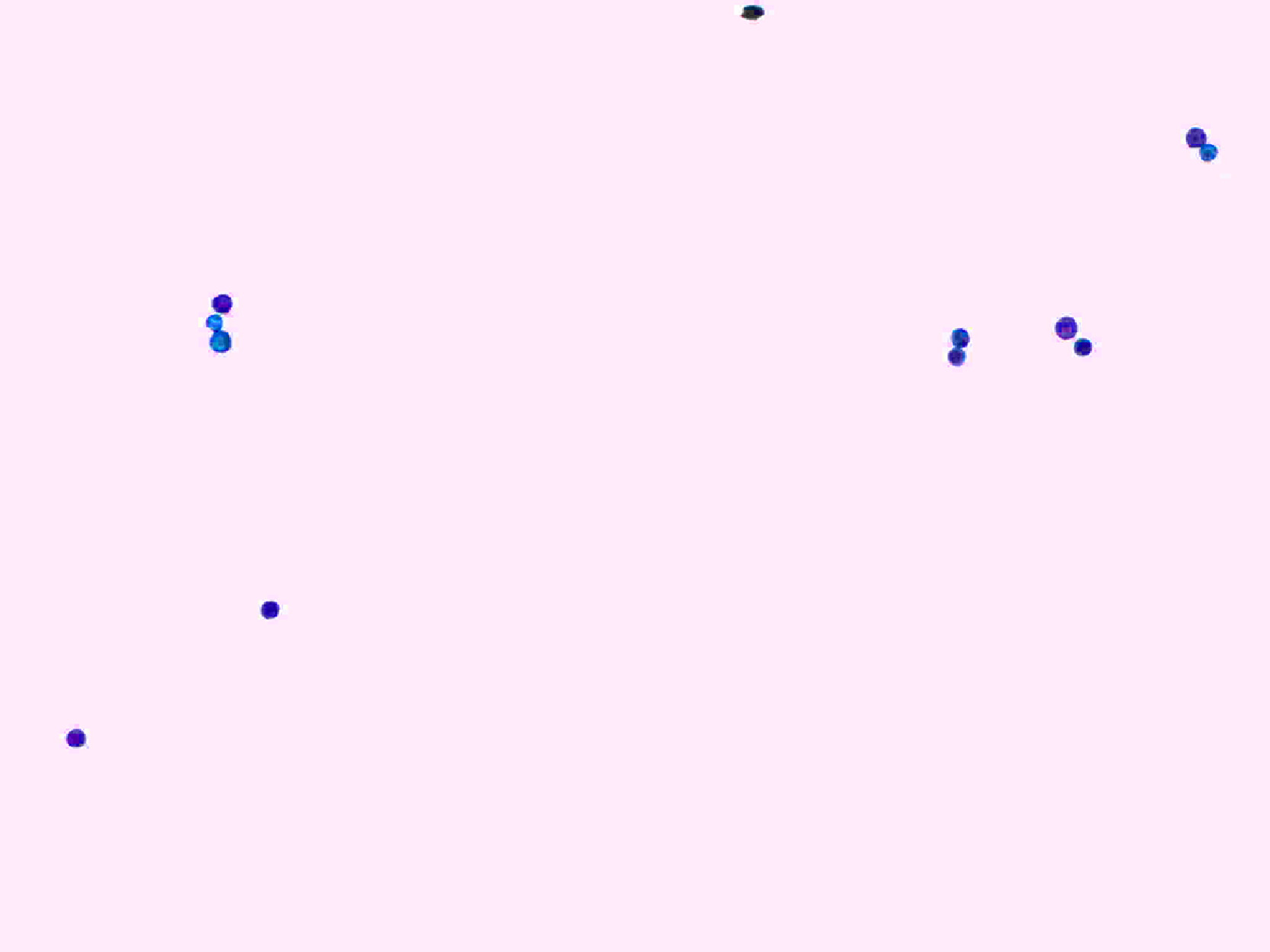

Supplement: Supplementary file 26 [file mmc26.jpg]

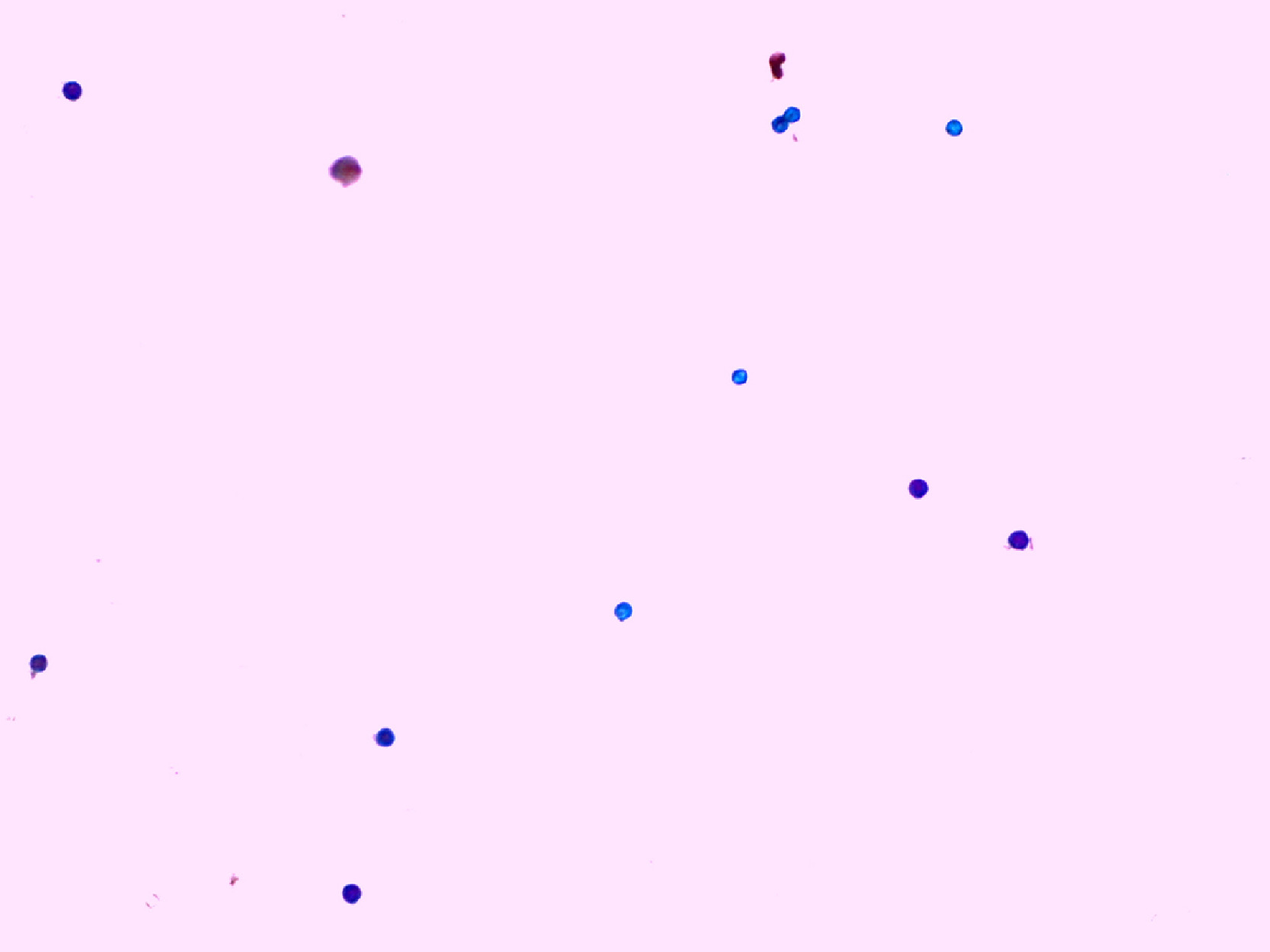

Supplement: Supplementary file 27 [file mmc27.jpg]

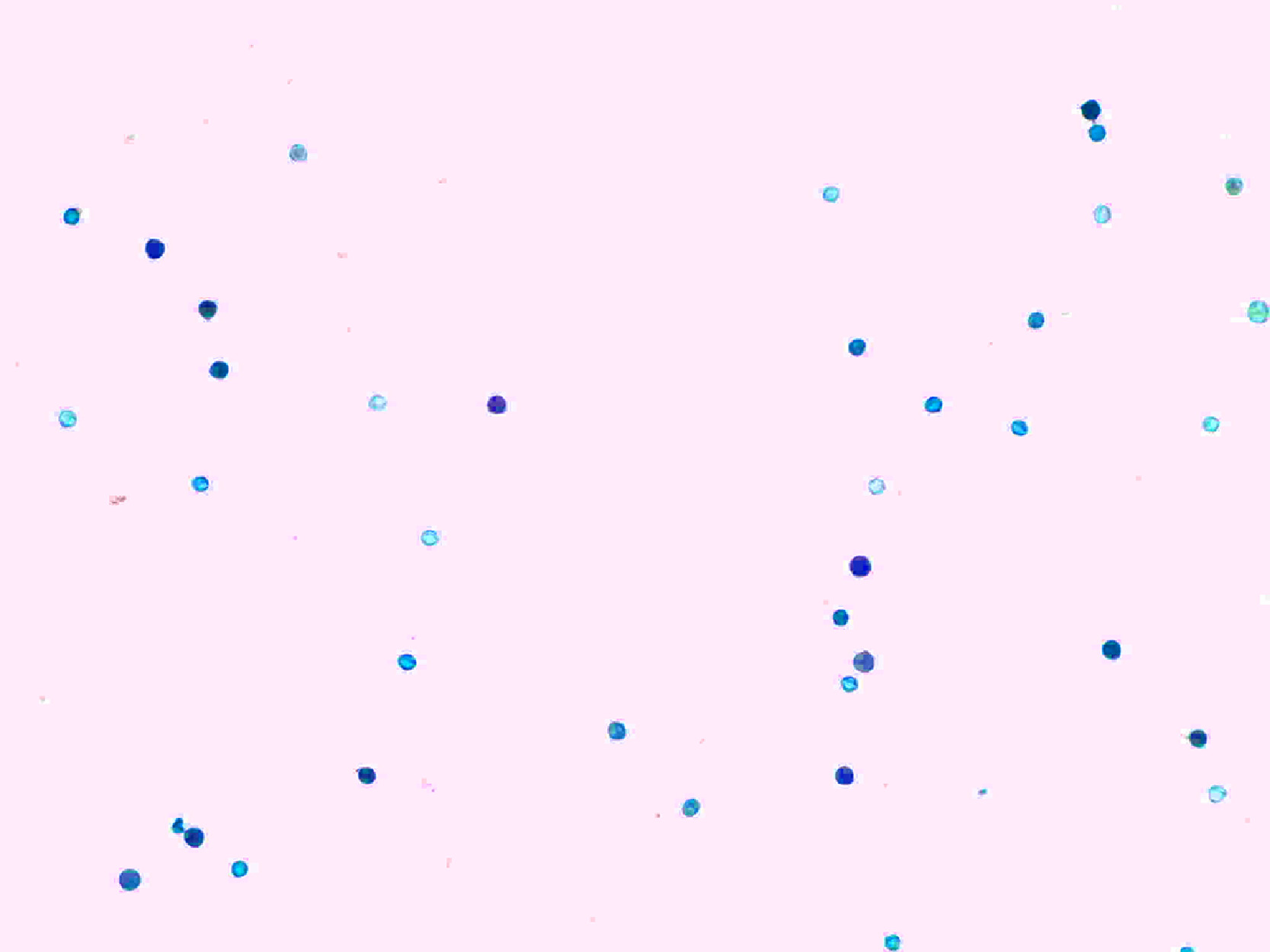

Supplement: Supplementary file 28 [file mmc28.jpg]

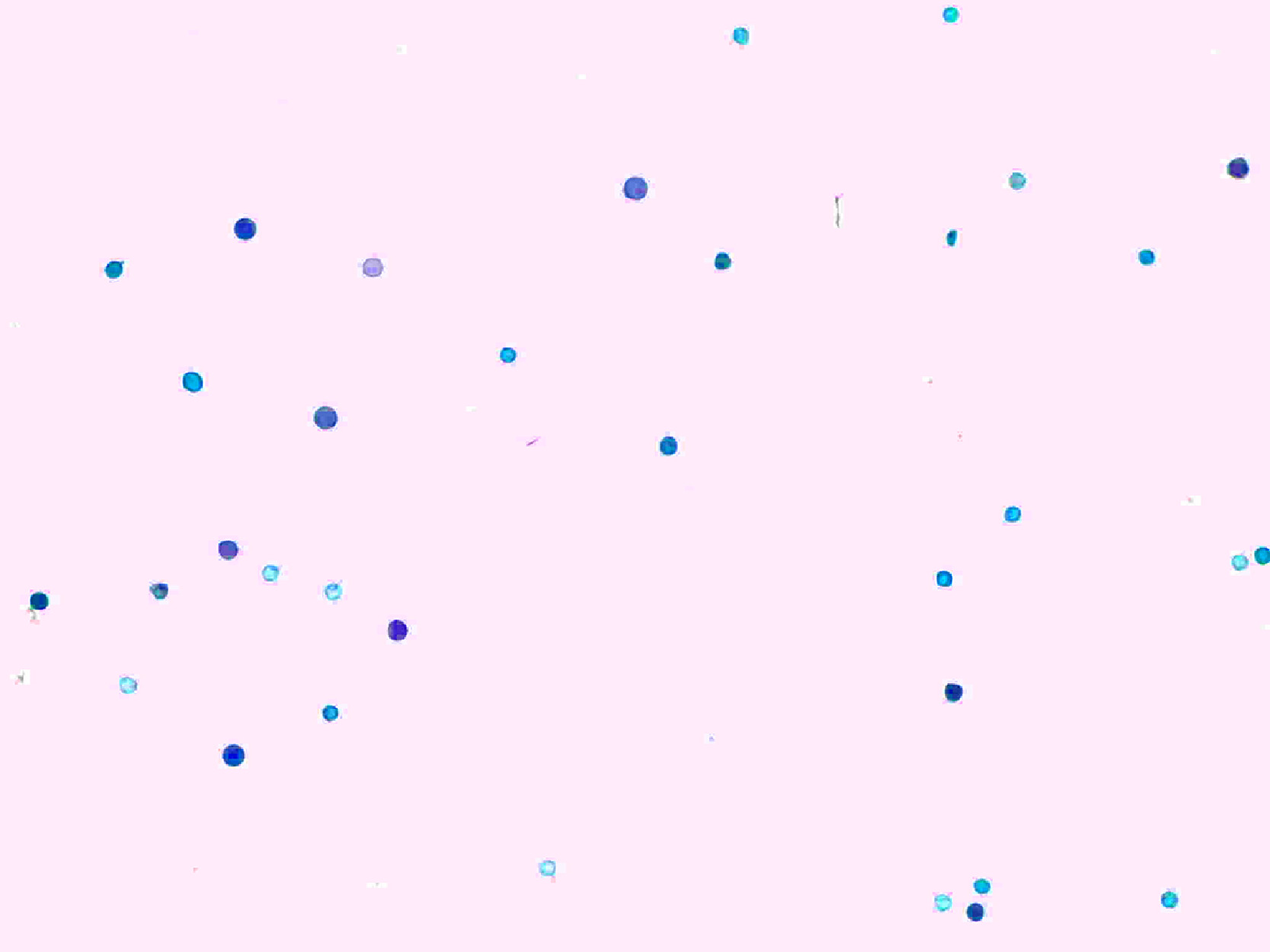

Supplement: Supplementary file 29 [file mmc29.jpg]

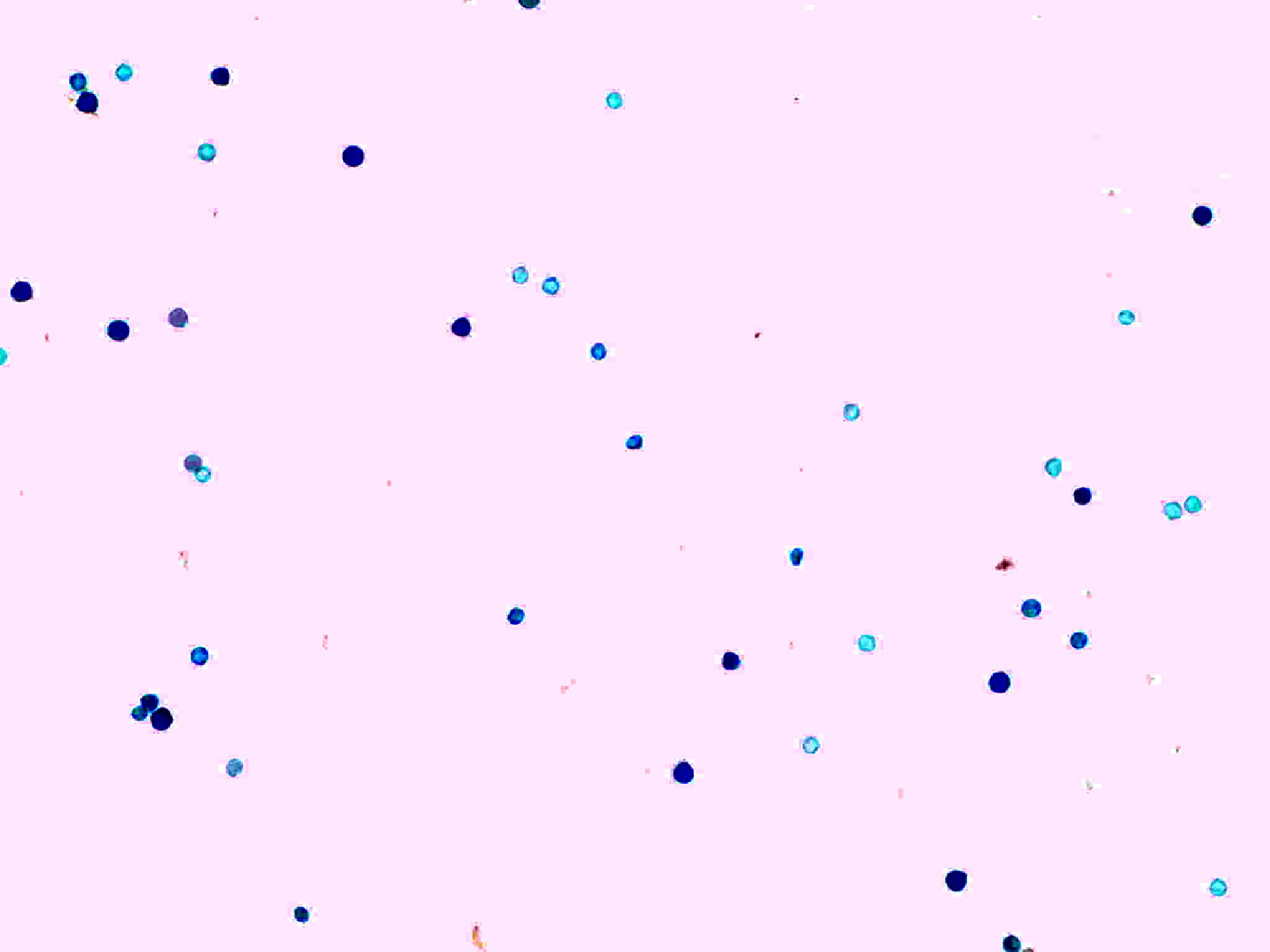

Supplement: Supplementary file 30 [file mmc30.jpg]

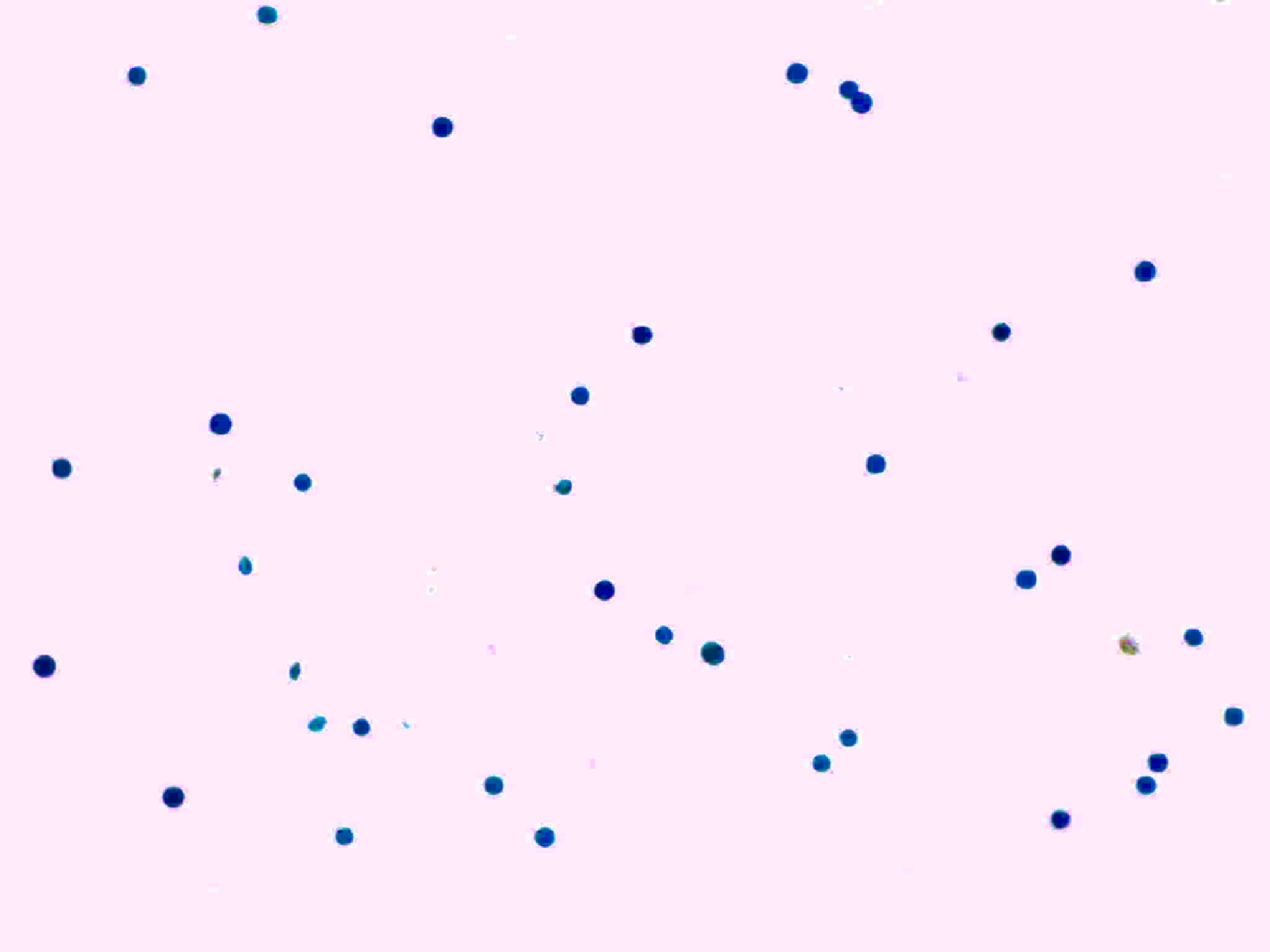

Supplement: Supplementary file 31 [file mmc31.jpg]
